# Rapsyn Homolog RPY-1 Modulates Locomotor Responses of *Caenorhabditis elegans* to Radial Extracorporeal Shock Waves

**DOI:** 10.3390/biomedicines14050960

**Published:** 2026-04-22

**Authors:** Tanja Hochstrasser, Leon Kaub, Christoph Schmitz

**Affiliations:** 1 Department of Anatomy II, Faculty of Medicine, Ludwig-Maximilians-University of Munich, 80336 Munich, Germany; leon.kaub@med.uni-muenchen.de (L.K.); christoph_schmitz@med.uni-muenchen.de (C.S.); 2 Physique des Cellules et Cancer, CNRS UMR168, Institut Curie, Sorbonne Université, Université PSL, 75005 Paris, France

**Keywords:** radial extracorporeal shock wave therapy, rESWT, neuromuscular junction, rapsyn, *rpy-1*, acetylcholine receptor clustering, *Caenorhabditis elegans*, *C. elegans*, locomotion, spasticity, mechanotransduction

## Abstract

**Background/Objectives**: Radial extracorporeal shock wave therapy (rESWT) is used to treat neuromuscular disorders such as spasticity, but the mechanisms by which rESWT modulates muscle tone remain incompletely understood. One proposed mechanism involves mechanical perturbation of the neuromuscular junction (NMJ), particularly destabilization of acetylcholine receptor (AChR) clusters in the postsynaptic membrane. Because *rapsyn* knockout mice are not viable, *Caenorhabditis elegans* (*C. elegans*) provides an alternative model for studying neuromuscular signaling, expressing the rapsyn homolog RPY-1, a postsynaptic scaffolding protein involved in AChR organization at the NMJ. This study examined whether loss of RPY-1 alters locomotor responses of *C. elegans* to radial extracorporeal shock wave (rESW) exposure. **Methods**: Wild-type worms and *rpy-1* knockout worms (*rpy-1*-KOs) were exposed to defined numbers of rESWs. Locomotor behavior was quantified using automated tracking of parameters describing speed, trajectory and body-wave dynamics. Behavioral responses were analyzed both as absolute values and relative to genotype-specific baseline values. **Results**: rESW exposure produced pronounced alterations in locomotor behavior across all parameters analyzed. Absolute values revealed baseline differences between genotypes. After normalization to genotype-specific baseline values, wild-type worms and *rpy-1*-KOs responded similarly to moderate exposure levels. At higher exposure levels, genotype-dependent differences became more apparent. Locomotor impairment was most pronounced immediately after exposure but improved during the subsequent recovery period of three hours. **Conclusions**: rESWs induced strong but largely reversible locomotor alterations in *C. elegans* during the first hours after exposure. Loss of the rapsyn homolog RPY-1 modified these responses, particularly at higher exposure levels. These findings indicate that RPY-1 influences behavioral responses to rESW exposure, while direct effects on NMJ structure or AChR organization cannot be determined from the present data.

## 1. Introduction

Radial extracorporeal shock wave therapy (rESWT) is widely used in clinical practice for the treatment of musculoskeletal disorders and neurological conditions associated with increased muscle tone, including spasticity in patients with cerebral palsy or stroke [1,2,3,4]. Radial extracorporeal shock waves (rESWs) are high-pressure acoustic impulses that propagate through biological tissues and induce transient mechanical perturbations at the cellular and extracellular levels [5,6,7]. Numerous clinical studies have demonstrated the beneficial effects of rESWT in reducing muscle spasticity and improving functional mobility [8,9,10]. However, the cellular and physiological mechanisms underlying these therapeutic effects remain incompletely understood.

One hypothesis that has gained increasing attention is that rESWs influence neuromuscular transmission by acting on the neuromuscular junction (NMJ) [11,12,13]. Experimental work in vertebrate models has shown that exposure to rESWs can affect NMJ structure and function. In particular, it has been demonstrated that rESWs applied to skeletal muscle can induce transient degeneration of NMJ components and temporary impairment of neuromuscular transmission, followed by gradual structural and functional recovery [11,12,13]. Structural analyses revealed alterations of the postsynaptic endplate region, including disruption of acetylcholine receptor (AChR) clusters and disorganization of junctional folds, suggesting that the postsynaptic apparatus may be sensitive to mechanical perturbation by rESWs [11,12].

Clinical findings are also compatible with a neuromuscular mechanism. In patients with cerebral palsy or stroke, rESWT has repeatedly been reported to reduce muscle tone and improve passive joint mobility [2,3,4,8,9,10]. The magnitude and time course of these effects can resemble those observed after focal chemodenervation therapies such as botulinum toxin type A injections [4]. Botulinum neurotoxins inhibit neuromuscular transmission by entering presynaptic motor nerve terminals and cleaving SNARE proteins required for synaptic vesicle fusion, thereby preventing the release of acetylcholine at the NMJ [14,15,16]. Although the molecular mechanisms differ, both interventions appear capable of transiently weakening neuromuscular transmission and thereby reducing pathological muscle overactivity.

The interpretation that rESWs influence neuromuscular transmission also raises the question of how the structural stability of the NMJ determines its susceptibility to mechanical perturbation. The stability of the postsynaptic membrane at the NMJ depends critically on the clustering and anchoring of AChRs. In vertebrates, this process is mediated by the scaffolding protein rapsyn (43 kDa receptor-associated protein of the synapse), which interacts with AChRs and cytoskeletal elements to stabilize receptor clusters within the postsynaptic membrane [17,18,19]. Mutations in *rapsyn* disrupt AChR clustering and cause congenital myasthenic syndromes in humans [20]. In animal models, complete loss of rapsyn prevents formation of functional NMJs and leads to severe neuromuscular dysfunction and perinatal lethality [17]. These findings illustrate the central importance of postsynaptic structural organization for efficient neuromuscular transmission.

The nematode *Caenorhabditis elegans* (*C. elegans*) provides a valuable model system for investigating molecular mechanisms of neuromuscular signaling [21,22,23]. The neuromuscular system of *C. elegans* shares key functional principles with vertebrate NMJs, including cholinergic motor neurons that activate body-wall muscle through receptor-mediated synaptic transmission [22,23]. Importantly, *C. elegans* expresses a homolog of rapsyn known as RPY-1, which participates in the clustering of AChRs at the NMJ [24]. Genetic disruption of *rpy-1* alters cholinergic neuromuscular signaling and produces phenotypes of *C. elegans* consistent with impaired AChR function at the NMJ [24].

Previous work has shown that exposure of *C. elegans* to rESWs results in significant changes in locomotor activity, including reductions in movement speed and alterations in body-wave parameters [7,25]. Because the entire organism is exposed to rESWs, both central and peripheral components of the motor system of *C. elegans* may be affected. Nevertheless, genetic manipulation of NMJ-associated proteins in *C. elegans* provides an opportunity to examine whether molecules involved in synaptic stabilization influence the response of the neuromuscular system to mechanical perturbation by rESWs. Furthermore, because *rapsyn* null mice are not viable beyond the neonatal period [17], *C. elegans* represents a unique experimental system in which the consequences of complete loss of a rapsyn-family protein can be studied in vivo.

Based on these considerations, the central hypothesis of the present study was that RPY-1 contributes to the structural stability of the NMJ and thereby influences the behavioral response of *C. elegans* to mechanical perturbation induced by rESWs. Specifically, we hypothesized that disruption of the *rpy-1* gene would alter the susceptibility of locomotor behavior to rESW-induced disturbances. To test this hypothesis, wild-type worms and *rpy-1* knockout worms (*rpy-1*-KOs) were exposed to defined numbers of rESWs, and locomotor behavior was quantified using automated tracking. In addition, fluorescence imaging of worms expressing an RPY-1::GFP fusion protein was performed to explore whether rESW exposure is associated with detectable changes in the abundance or localization of this postsynaptic scaffolding protein in vivo. To our knowledge, this is the first study examining how loss of a rapsyn-related postsynaptic scaffold protein influences the behavioral response of an otherwise intact neuromuscular system to rESW exposure.

Of particular relevance in this context are recent observations indicating that mechanical perturbations of the nervous system can produce both acute and delayed functional consequences [26]. In a recent study investigating the effects of both blast-related shock waves and rESW exposure on *C. elegans* (using the same rESW generator as in the present study, but a slightly different exposure protocol), worms exhibited an immediate impairment of locomotor behavior followed by recovery within one day, whereas longer-term deterioration occurred later and was associated with progressive neuronal dysfunction [26]. As this study is currently available as a preprint, it should be interpreted with appropriate caution. Our experiments were designed to examine short-term locomotor effects of rESW exposure and the contribution of the rapsyn homolog RPY-1 during the first hours after exposure. Accordingly, the present study did not address potential delayed effects occurring at later time points.

## 2. Materials and Methods

Wild-type *C. elegans* N2 worms and the *rpy-1*(ok145) knockout strain NM1581 were obtained from the Caenorhabditis Genetics Center (Minneapolis, MN, USA). The NM1581 strain was generated as part of the *C. elegans* Gene Knockout Project at the Oklahoma Medical Research Foundation and distributed through the International *C. elegans* Gene Knockout Consortium [27]. For fluorescence imaging experiments, the transgenic strain N2; Ex(Prpy-1::rpy-1::GFP + rol-6), expressing an RPY-1::GFP fusion protein under control of the endogenous *rpy-1* promoter, was used. This strain was kindly provided by Prof. Junho Lee (Institute of Molecular Biology and Genetics, Seoul National University, Seoul, Republic of Korea).

All worms were maintained according to standard procedures for *C. elegans* culture [28]. Animals were grown on nematode growth medium (NGM) agar plates seeded with *Escherichia coli* OP50 as a food source and kept at 20 °C. The NGM plates consisted of 50 mM NaCl (Carl Roth, Karlsruhe, Germany), 0.25% Bacto-Peptone (BD Biosciences, Franklin Lakes, NJ, USA), 1.8% agar (BD Biosciences, Franklin Lakes, NJ, USA), 1 mM MgSO_4_ (Carl Roth, Karlsruhe, Germany), 1 mM KH_2_PO_4_ (pH 6; Merck, Darmstadt, Germany), 1 mM CaCl_2_ (Carl Roth, Karlsruhe, Germany) and 5 µg/mL cholesterol (Sigma-Aldrich, St. Louis, MO, USA) dissolved in ethanol. To obtain synchronized populations, gravid adult worms were treated with alkaline hypochlorite solution consisting of 250 mM NaOH (Carl Roth, Karlsruhe, Germany) and 20% sodium hypochlorite (Thermo Fisher Scientific, Waltham, MA, USA). Surviving embryos were washed twice in M9 buffer containing 21 mM Na_2_HPO_4_ (Sigma-Aldrich, St. Louis, MO, USA), 22 mM KH_2_PO_4_ (Merck, Darmstadt, Germany), 85 mM NaCl (Carl Roth, Karlsruhe, Germany) and 1 mM MgSO_4_ (Carl Roth, Karlsruhe, Germany), and allowed to hatch overnight at 20 °C with gentle agitation. The resulting synchronized L1 larvae were transferred to fresh NGM plates seeded with OP50 and cultured until young adulthood. Approximately 48 h after plating, young adult worms were collected in M9 buffer for the experiments.

Radial extracorporeal shock waves were applied using a clinically established rESWT device (Swiss DolorClast, Electro Medical Systems, Nyon, Switzerland). The device was equipped with a 6 mm applicator and operated at an air pressure of 2 bar and a pulse frequency of 5 Hz. To ensure standardized exposure conditions, the handpiece was mounted vertically on a drill stand (Wolfcraft, Kempenich, Germany). A 5.5 × 2 mm O-ring (C. Otto Gehrckens GmbH, Pinneberg, Germany) was placed around the applicator tip to maintain a defined distance between the applicator and the experimental sample. Worm suspensions were prepared in M9 buffer, and 300 µL aliquots were dispensed into individual wells of a U-bottom 96-well plate (VWR International, Radnor, PA, USA). The applicator of the rESWT device was lowered into a well until the plate was held firmly in place, allowing consistent delivery of rESWs to the worm suspension as previously described [7,25].

Two complementary experimental paradigms were used to investigate the behavioral effects of rESW exposure. In the first experiment, a dose–response design was implemented to examine the effect of increasing numbers of rESWs. Worms of both genotypes were exposed to either 0, 100 or 300 rESWs. Immediately after exposure, the worms were transferred to food-free NGM plates for behavioral recording. The selected exposure levels were defined to represent moderate- and higher-intensity conditions within the operational range previously applied with the rESW setup in *C. elegans* [7,25]. In the second experiment, the temporal dynamics of behavioral responses were assessed in a recovery design. In this series, worms were exposed to either 0 or 300 rESWs and then allowed to recover in the wells for defined time intervals of 0, 30 or 180 min before behavioral analysis.

For behavioral analysis, worms were transferred from the wells to NGM plates using a filtration-based transfer method that minimizes mechanical disturbance [7,25]. To prevent worms from adhering to pipette tips, 30 µL of M9 buffer containing 1% Tween-20 (Sigma-Aldrich, St. Louis, MO, USA) was added to each well prior to transfer. The worm suspension was pipetted onto a polyethersulfone membrane mounted on a vacuum filtration device (47 mm diameter, 0.22 µm pore size; Merck Millipore, Darmstadt, Germany). The membrane containing the worms was then inverted onto a food-free NGM plate and removed immediately before recording. Behavioral recordings were obtained using the WormLab imaging system (MBF Bioscience, Williston, VT, USA). Plates were positioned to keep the worms within the camera field of view. Video acquisition was performed with a 20-megapixel monochrome digital camera (acA5472-17um, Basler, Ahrensburg, Germany) equipped with a 60 mm macro lens (AF Micro-Nikkor 60 mm 1:2.8D; Nikon, Tokyo, Japan). Videos were recorded at 7.5 frames per second for 60 s, resulting in 450 frames per recording. Images were captured at a resolution of 5484 × 3660 pixels.

Video sequences were analyzed using WormLab software (version 2022.1.1, MBF Bioscience, Williston, VT, USA). Individual worms were tracked automatically across the entire recording period, and locomotor parameters were extracted for quantitative analysis. Only worms with a body length greater than 650 µm and continuous tracking for at least 30 s (≥225 frames) were included. For each worm, five behavioral parameters were calculated: (i) peristaltic speed, defined as the total peristaltic track length divided by time; (ii) total track length, representing the cumulative distance traveled during the recording; (iii) body-wave wavelength, defined as twice the distance between the positive and negative inflection points of the body wave; (iv) reversal frequency, representing the occurrence of transitions into backward movement; and (v) mean body-wave amplitude, defined as the average centroid displacement along the body axis. These parameters collectively characterize the speed, trajectory and body-wave dynamics of worm locomotion [29], and have been widely used as quantitative indicators of neuromuscular function in *C. elegans* (e.g., [30,31,32]).

Initial inspection of the datasets revealed pronounced baseline differences in locomotor behavior between wild-type worms and *rpy-1*-KOs. Because these differences reflect intrinsic genotype-dependent locomotor characteristics rather than effects of rESW exposure, behavioral responses to rESWs were analyzed as values normalized to genotype-specific control groups. For each locomotor parameter, measurements from exposed worms were expressed relative to the mean value of the corresponding unexposed group of the same genotype. This normalization ensured that statistical analyses focused on exposure-induced changes in locomotor behavior rather than baseline genotype differences. Absolute values were analyzed and presented separately to document the baseline phenotype and to complement the normalized analyses.

All statistical analyses were performed in Python (versions 3.9.18; Python Software Foundation, Wilmington, DE, USA) using the libraries numpy, scipy, pandas and statsmodels. Normality within experimental groups was assessed using the Shapiro–Wilk test (implemented in scipy), which indicated that assumptions for parametric analysis of variance were not satisfied. Thus, non-parametric methods were applied.

In the first experimental series, involving two experimental factors (genotype and rESW exposure), group differences were evaluated using the Scheirer–Ray–Hare test, a non-parametric extension of two-way analysis of variance based on ranked data [33]. This analysis tested the main effects of genotype and exposure as well as their interaction. When significant effects were detected, post hoc pairwise comparisons between genotype–exposure groups were performed using Dunn’s test with Holm correction for multiple testing [34].

In the second experimental series, which included three experimental factors (genotype, rESW exposure, recovery time), a rank-based factorial approach was applied. Observations were converted to ranks and analyzed using an ordinary least squares model implemented in Python’s statsmodels library. The resulting factorial analysis of variance table based on ranked data was used to evaluate main effects and interactions among genotype, exposure and recovery time. In view of the partially crossed design of this experiment, recovery-related effects were interpreted primarily on the basis of biologically relevant pairwise comparisons in addition to the omnibus analysis. Significant effects were followed by pairwise comparisons using Dunn-type tests with Holm correction [34].

Statistical significance was defined as *p* < 0.05. Detailed descriptions of all statistical analyses, full descriptive statistics and exact *p*-values are provided in Supplementary File S1.

To examine whether rESW exposure affected the localization of the RPY-1 protein in vivo, fluorescence imaging experiments were performed using worms expressing the RPY-1::GFP fusion protein. Worms were mounted on agarose pads (8–10%) prepared on microscope slides and immobilized in M9 buffer containing a mounting mixture of 2% levamisole hydrochloride and 50% nanosphere size standards. Samples were covered with a coverslip and imaged using an Olympus IXplore SpinSR confocal microscope (Olympus, Tokyo, Japan) equipped with a UplanS Apo 60×/1.30 silicon oil objective. GFP fluorescence was excited at 488 nm. Image acquisition was performed as z-stacks with 0.5 µm increments, and maximum intensity projections were generated by collapsing the z-stacks. Worms were imaged either immediately after exposure to 300 rESWs or after a recovery interval of 60 min. The resulting images were evaluated qualitatively.

## 3. Results

### 3.1. Absolute Values, and Baseline Locomotor Differences Between Wild-Type Worms and Rpy-1-KOs

**Figure 1:**
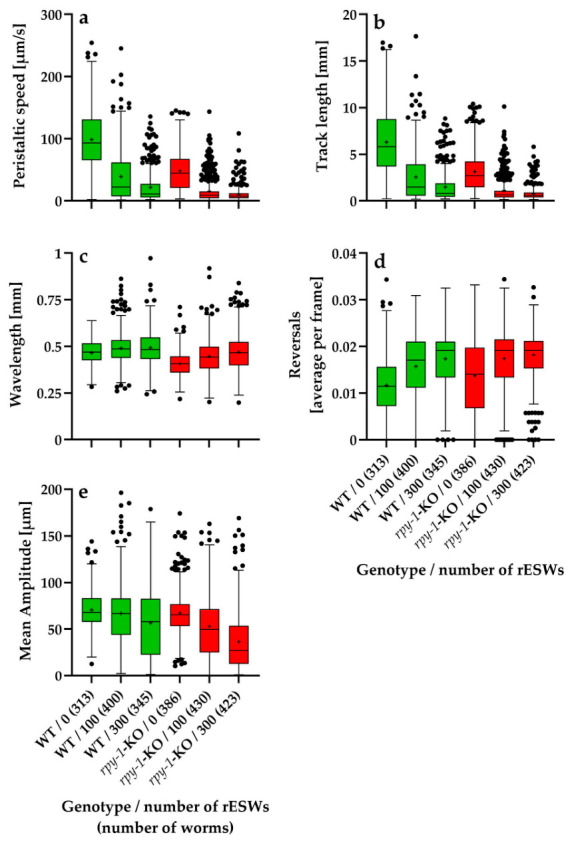
Tukey boxplots showing the absolute values of five locomotion parameters—peristaltic speed (**a**), track length (**b**), wavelength (**c**), reversal frequency (**d**) and mean amplitude (**e**)—in wild-type *C. elegans* N2 worms (WT; green) and the *rpy-1*(ok145) knockout strain NM1581 (*rpy-1*-KO; red), plotted as a function of the number of rESWs administered in the first experimental series. Sample sizes (numbers in parentheses) ranged from 313 worms (N2, 0 rESWs) to 430 worms (*rpy-1*-KO, 100 rESWs).

**Figure 2:**
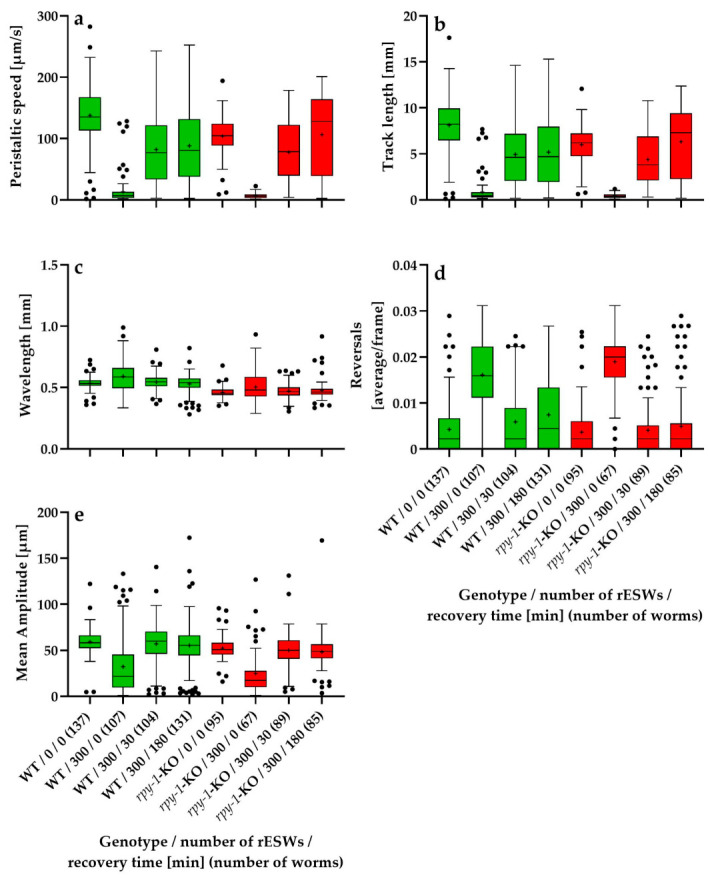
Tukey boxplots showing the absolute values of five locomotion parameters—peristaltic speed (**a**), track length (**b**), wavelength (**c**), reversal frequency (**d**) and mean amplitude (**e**) –in wild-type *C. elegans* N2 worms (WT; green) and the *rpy-1*(ok145) knockout strain NM1581 (*rpy-1*-KO; red), plotted as a function of the number of rESWs administered and the recovery time after rESW exposure in the second experimental series. Sample sizes ranged from 67 worms (*rpy-1*-KO, 300 rESWs, 0 min recovery) to 137 worms (WT, 0 rESWs, 0 min recovery).

Figure 1 and Figure 2 summarize the absolute values of five locomotion parameters—peristaltic speed, track length, wavelength, reversal frequency and mean amplitude—in wild-type *C. elegans* N2 worms and the *rpy-1*-KOs; data were obtained in the first experimental series (Figure 1) and the second experimental series (Figure 2) of this study.

Baseline locomotor behavior differed markedly between wild-type worms and *rpy-1*-KOs. Across the analyzed locomotor parameters, *rpy-1*-KOs exhibited altered movement patterns affecting both forward locomotion and body-wave dynamics. Mutants showed reduced peristaltic speed and shorter track lengths compared with wild-type worms (Figure 1a,b and Figure 2a,b). Differences were also observed in body-wave morphology, including wavelength and mean body-wave amplitude (Figure 1c,e and Figure 2c,e). Together, these results indicate that loss of the rapsyn homolog RPY-1 impaired neuromuscular performance under baseline conditions.

Because these genotype-dependent baseline differences reflect intrinsic locomotor characteristics rather than responses to rESW exposure, the analysis of rESW-induced effects was performed using relative values normalized to the corresponding genotype-specific control groups. This normalization procedure allowed the statistical analysis to focus specifically on exposure-induced behavioral changes. At the same time, the absolute values shown in Figure 1 and Figure 2 document the baseline locomotor phenotype and thereby complement the normalized analyses.

### 3.2. Dose-Dependent Effects of rESW Exposure on Relative Locomotor Behavior

**Figure 3:**
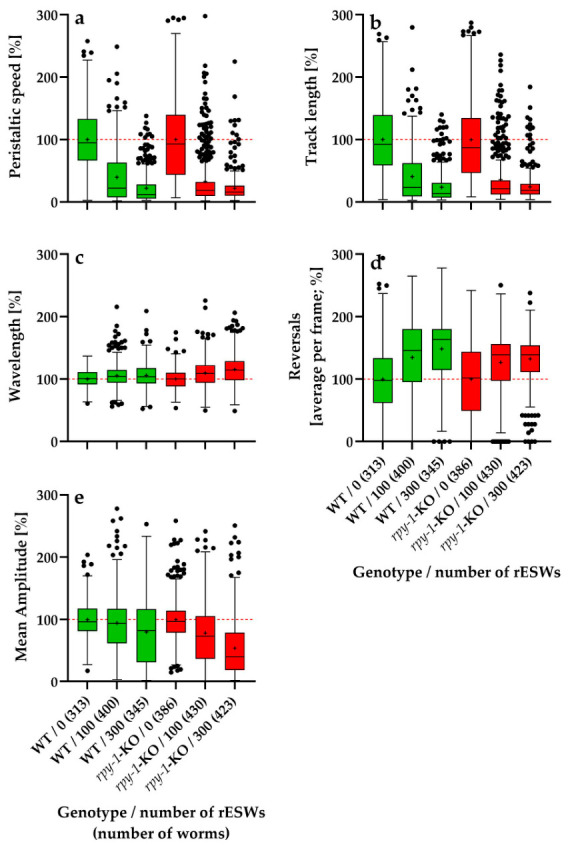
Tukey boxplots of relative data (normalized to the corresponding genotype-specific control groups) of peristaltic speed (**a**), track length (**b**), wavelength (**c**), reversal frequency (**d**) and mean amplitude (**e**) for wild-type *C. elegans* N2 worms (WT; green) and the *rpy-1*(ok145) knockout strain NM1581 (*rpy-1*-KO; red), plotted as a function of the number of rESWs administered in the first experimental series. Each box summarizes data from 313 (N2, 0 rESWs) to 430 (*rpy-1*-KO, 100 rESWs) individual worms. In each panel, 100% (the normalized mean value of the genotype-specific control group) is indicated by a horizontal dashed red line.

Across all parameters, exposure to rESWs induced clear behavioral changes, but the direction and magnitude of the genotype effect depended on the locomotor parameter examined (Figure 3).

The results of the statistical analysis of the data shown in Figure 3 (*p* values) are summarized in Table 1.

#### 3.2.1. Relative Peristaltic Speed

Relative peristaltic speed decreased markedly with increasing rESW exposure in both genotypes (Figure 3a). In wild-type worms, the mean normalized peristaltic speed declined from 100% in controls to approximately 39.7% after 100 rESWs and 22.2% after 300 rESWs. In *rpy-1*-KOs, the corresponding values were approximately 32.9% after 100 rESWs and 21.8% after 300 rESWs. At 100 rESWs, wild-type worms and *rpy-1*-KOs did not differ significantly. Although the mean value was slightly lower in the mutant, the distributions overlapped substantially, indicating similar susceptibility to moderate exposure. At 300 rESWs, a statistically significant genotype difference emerged. Descriptively, the mean values were very similar, but the rank distributions differed slightly, with wild-type worms showing lower values than *rpy-1*-KOs. Thus, the genotype effect at 300 rESWs reflected a subtle shift in distribution rather than a clear separation of group means. These data suggest that RPY-1 may modulate the response to strong mechanical perturbation, though not in a strictly directional manner.

#### 3.2.2. Relative Track Length

Relative track length showed a pattern similar to that of relative peristaltic speed (Figure 3b). In wild-type worms, normalized track length decreased from 100% in controls to approximately 40.6% after 100 rESWs and 23.6% after 300 rESWs. In *rpy-1*-KOs, the corresponding values were approximately 35.8% and 24.2%. At 100 rESWs, no statistically significant difference was detected between genotypes. Although *rpy-1*-KOs showed slightly lower mean values, moderate exposure reduced locomotor output similarly in both groups. At 300 rESWs, however, a significant genotype difference emerged. As for relative peristaltic speed, the difference was modest at the level of group means but evident in the rank distributions, with wild-type worms showing slightly lower normalized values. Thus, RPY-1 influenced the response to high exposure levels, but the effect did not simply reflect increased susceptibility of the mutant.

#### 3.2.3. Relative Wavelength

Relative wavelength behaved differently from relative peristaltic speed and relative track length (Figure 3c). In wild-type worms, normalized wavelength increased slightly to approximately 105.3% after 100 rESWs and 105.8% after 300 rESWs. In *rpy-1*-KOs, the increase was larger, reaching approximately 109.9% and 115.5%, respectively. At 100 rESWs, the difference between wild-type worms and *rpy-1*-KOs was already statistically significant. At 300 rESWs, this difference became more pronounced, with *rpy-1-*KOs showing the strongest upward shift. Thus, absence of *rpy-1* was associated with a stronger exposure-induced increase in wavelength, suggesting that RPY-1 influenced the stability of body-wave geometry under mechanical stress induced by rESWs.

#### 3.2.4. Relative Reversal Frequency

Relative reversal frequency increased with rESW exposure in both genotypes (Figure 3d). In wild-type worms, the mean value rose from 100% to approximately 134.8% after 100 rESWs and 148.6% after 300 rESWs. In *rpy-1*-KOs, the increase was smaller, reaching approximately 126.8% and 132.5%. At 100 rESWs, the difference between genotypes was not significant. At 300 rESWs, however, wild-type worms exhibited a significantly stronger increase in reversal frequency than *rpy-1*-KOs. Thus, for this parameter the absence of *rpy-1* attenuated rather than enhanced the behavioral response to strong rESW exposure, indicating that the influence of RPY-1 was parameter-specific.

#### 3.2.5. Relative Mean Body-Wave Amplitude

Relative mean body-wave amplitude showed the clearest genotype effect (Figure 3e). In wild-type worms, normalized amplitude was approximately 94.7% after 100 rESWs and decreased to 80.3% after 300 rESWs. In *rpy-1*-KOs, the reduction was substantially stronger, reaching approximately 78.6% and 54.3%, respectively. At 100 rESWs, the difference between genotypes was already statistically significant. At 300 rESWs, the difference became even more pronounced, with mutants showing the strongest reduction of all groups. These findings indicate that loss of *rpy-1* was associated with a markedly stronger effect of mechanical perturbation on body-wave amplitude, consistent with a role of RPY-1 in stabilizing neuromuscular output.

#### 3.2.6. Overall Pattern in the First Experimental Series

Overall, rESW exposure affected all locomotor parameters analyzed, but genotype effects depended on both exposure intensity and behavioral endpoint. At 100 rESWs, genotype differences were absent for peristaltic speed, track length and reversal frequency but present for wavelength and mean amplitude. At 300 rESWs, significant genotype differences occurred for all parameters. However, the direction of these effects varied. Specifically, *rpy-1*-KOs showed stronger responses for wavelength and especially mean amplitude, whereas wild-type worms showed a stronger increase in reversal frequency. For peristaltic speed and track length, genotype differences were statistically significant but descriptively modest. These findings indicate that RPY-1 modulated the behavioral response to rESW exposure in a dose-dependent and parameter-specific manner.

### 3.3. Recovery Dynamics of Relative Locomotor Behavior Within the First 180 Minutes After Exposure to 300 rESWs

**Figure 4:**
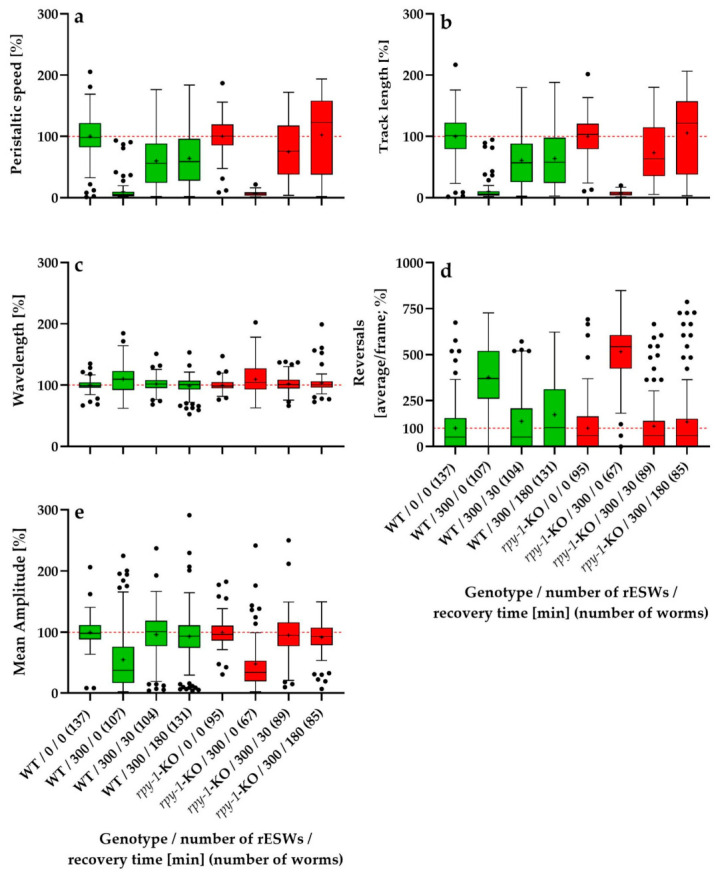
Tukey boxplots of relative data (normalized to the corresponding genotype-specific control groups) of peristaltic speed (**a**), track length (**b**), wavelength (**c**), reversals (**d**) and mean amplitude (**e**) for wild-type *C. elegans* N2 worms (WT; green) and the *rpy-1*(ok145) knock-out strain NM1581 (*rpy-1*-KO; red) as a function of the number of rESWs to which the worms were exposed and the recovery time after rESW exposure. Each box summarizes data from 67 (*rpy-1*-KO/300/0 min recovery) to 137 (WT/0 rESWs/0 min recovery) individual worms. In each panel, 100% (the normalized mean value of the genotype-specific control group) is indicated by a horizontal dashed red line.

Overall, exposure to 300 rESWs produced a pronounced immediate disturbance of locomotor behavior in both genotypes. However, the subsequent recovery trajectories differed among the locomotor parameters and, in some cases, also between wild-type worms and *rpy-1*-KOs (Figure 4).

The results of the statistical analysis of the data shown in Figure 4 (*p* values) are summarized in Table 2. In view of the partially crossed design of this experiment, the recovery data are most appropriately interpreted using the biologically relevant pairwise comparisons shown in Figure 4 and Table 2, rather than the omnibus main effect of the recovery factor alone. Such outcomes, including *p*-values of 1.000, can occur in rank-based factorial analyses, depending on how ranks are distributed across factor levels.

#### 3.3.1. Relative Peristaltic Speed

Relative peristaltic speed showed a pronounced immediate reduction after exposure to 300 rESWs in both genotypes, followed by progressive recovery (Figure 4a). In wild-type worms, normalized speed decreased from 100% in controls to approximately 9.8% immediately after exposure, then recovered to 59.8% after 30 min and 64.0% after 180 min. In *rpy-1*-KOs, the corresponding values were 6.7% immediately after exposure, 74.8% after 30 min and 102.4% after 180 min. Immediately after exposure, no statistically significant difference between genotypes was detected. Both groups exhibited a near-complete suppression of locomotor speed, and the distributions overlapped extensively. At 30 min, a genotype difference was still absent, although the descriptive values already suggested a more advanced recovery in *rpy-1*-KOs. At 180 min, a statistically significant genotype difference emerged. Wild-type worms recovered only to approximately two thirds of their baseline speed, whereas *rpy-1*-KOs returned to or slightly exceeded baseline levels. Thus, although both genotypes were severely affected acutely, the mutant showed a more complete relative recovery.

#### 3.3.2. Relative Track Length

Relative track length showed a pattern closely paralleling that of relative peristaltic speed (Figure 4b). In wild-type worms, normalized track length declined from 100% to approximately 10.5% immediately after exposure, then recovered to 60.9% after 30 min and 63.9% after 180 min. In *rpy-1*-KOs, the corresponding values were 7.5%, 73.3% and 105.5%. Immediately after exposure, no significant genotype difference was detected, indicating that the acute suppression of overall locomotor output was comparable in both groups. After 30 min, genotype differences remained non-significant, although the descriptive means indicated a stronger recovery in *rpy-1*-KOs. After 180 min, a statistically significant difference emerged. Wild-type worms remained at approximately 63.9% of baseline, whereas *rpy-1*-KOs recovered to approximately 105.5%. As for relative peristaltic speed, these findings indicate that the absence of *rpy-1* did not exacerbate the acute response but was associated with a more complete relative recovery during the later phase of observation.

#### 3.3.3. Relative Wavelength

Relative wavelength behaved differently from relative peristaltic speed and relative track length (Figure 4c). In wild-type worms, normalized wavelength increased to approximately 110.1% immediately after exposure, and then declined to 101.8% after 30 min and 98.6% after 180 min. In *rpy-1*-KOs, the corresponding values were 109.6%, 102.4% and 103.3%. Immediately after exposure, both genotypes showed a comparable increase in wavelength and did not differ significantly. At 30 min, wavelength values in both groups had already returned close to baseline, again without genotype differences. At 180 min, wild-type worms had essentially returned to baseline and *rpy-1*-KOs remained only slightly above baseline, but the difference remained statistically non-significant. Thus, rESW exposure produced a modest and transient increase in wavelength that was largely independent of RPY-1.

#### 3.3.4. Relative Reversal Frequency

Relative reversal frequency showed the most pronounced immediate increase in all locomotor parameters (Figure 4d). In wild-type worms, normalized reversal frequency increased from 100% to approximately 376.1% immediately after exposure, then decreased to 137.7% after 30 min and 173.5% after 180 min. In *rpy-1*-KOs, the corresponding values were 516.0%, 111.3% and 134.5%. Immediately after exposure, a statistically significant genotype difference was present, with *rpy-1*-KOs showing a markedly stronger increase in reversal frequency. At 30 min, this difference disappeared as both groups recovered substantially. The same was observed at 180 min, when reversal frequency remained somewhat elevated but did not differ significantly between genotypes. Thus, absence of RPY-1 amplified the acute behavioral disturbance but did not alter subsequent short-term recovery.

#### 3.3.5. Relative Mean Body-Wave Amplitude

Relative mean body-wave amplitude showed a pronounced immediate decrease after exposure to 300 rESWs in both genotypes, followed by rapid recovery (Figure 4e). In wild-type worms, normalized amplitude declined from 100% to approximately 54.5% immediately after exposure, and then recovered to 96.4% after 30 min and 93.5% after 180 min. In *rpy-1*-KOs, the corresponding values were 47.4%, 95.4%, and 92.0%. Immediately after exposure, no statistically significant genotype difference was detected, despite slightly lower descriptive values in the mutant group. At 30 min, both genotypes had returned to values close to baseline and remained similar to each other. The same pattern persisted at 180 min, indicating that the reduction in body-wave amplitude induced by rESW exposure was strong but rapidly reversible and largely independent of RPY-1 during the short-term recovery period.

#### 3.3.6. Overall Pattern in the Second Experimental Series

Overall, exposure to 300 rESWs produced a strong immediate disturbance of locomotor behavior in all parameters analyzed (Figure 4), followed by varying degrees of recovery during the subsequent 180 min. However, recovery trajectories differed among parameters and were only partly influenced by genotype. Relative peristaltic speed and relative track length showed severe immediate suppression in both genotypes and significant genotype differences only after 180 min, when *rpy-1*-KOs had recovered more completely than wild-type worms. Relative wavelength showed only modest deviations from baseline and no genotype differences at any time point. Relative reversal frequency displayed the strongest acute response, with *rpy-1*-KOs showing a greater immediate increase than wild-type worms, although this difference disappeared during recovery. Relative mean body-wave amplitude showed a strong acute decrease followed by near-complete normalization within 30 min in both genotypes. Taken together, these results indicate that the absence of *rpy-1* did not produce a uniform pattern of increased or prolonged impairment after exposure to 300 rESWs. Instead, the influence of RPY-1 on the short-term response was parameter-specific, affecting the magnitude of acute disturbances in some cases and the dynamics of recovery in others.

### 3.4. Localization of RPY-1 Following rESW Exposure

**Figure 5:**
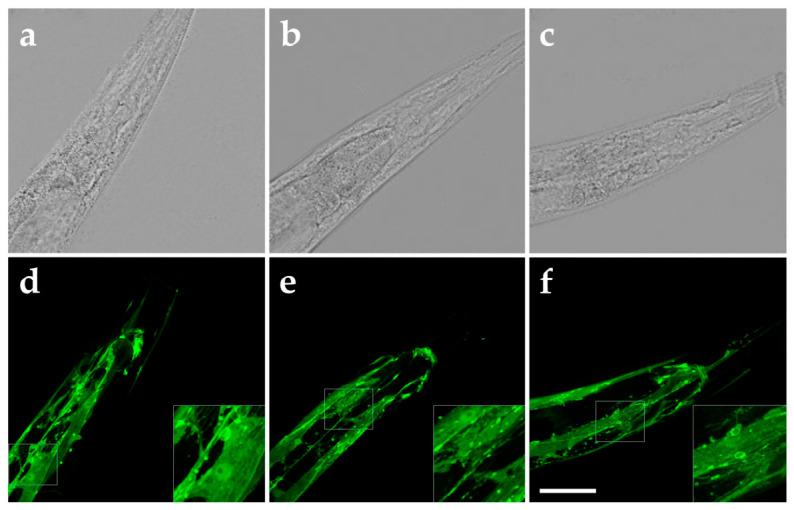
Brightfield photomicrographs (**a**–**c**) and GFP fluorescence photomicrographs (**d**–**f**) of N2; Ex(Prpy-1::rpy-1::GFP + rol-6) worms, expressing an RPY-1::GFP fusion protein under control of the endogenous *rpy-1* promoter, without exposure to rESWs (**a**,**d**), immediately after exposure to 300 rESWs (**b**,**e**) and 60 min after exposure to 300 rESWs. Scale bars, 25 µm, inset: 10 µm.

To explore whether rESW exposure affected the abundance or localization of RPY-1 in vivo, transgenic worms expressing an RPY-1::GFP fusion protein were examined by confocal microscopy. In unexposed worms, RPY-1::GFP fluorescence was detected in body-wall muscles and neurons, consistent with the expected distribution of the protein (Figure 5a,d).

Exposure to 300 rESWs did not reveal obvious changes in the overall pattern of RPY-1::GFP localization upon qualitative inspection. Both immediately after exposure (Figure 5b,e) and after a recovery period of 60 min (Figure 5c,f), the distribution of fluorescence signals appeared largely unchanged compared with unexposed controls. In some worms, a slight increase in diffuse or nonspecific fluorescence signals was observed following exposure, but no systematic alterations in RPY-1 localization or abundance were detected by qualitative inspection.

These observations indicate that rESW exposure did not produce overt redistribu-tion of RPY-1 on the timescale examined. However, because these imaging data are exploratory and qualitative, they do not allow definitive conclusions regarding subtle changes in protein abundance, localization, or nanoscale organization.

## 4. Discussion

The present study demonstrated that exposure of *C. elegans* to rESWs produced pronounced alterations in locomotor behavior. Worms lacking the rapsyn homolog RPY-1 exhibited a clear locomotor deficit already under baseline conditions, confirming earlier reports that this protein contributes to normal neuromuscular function in *C. elegans* [24]. When worms were exposed to rESWs, substantial behavioral changes were observed in both genotypes. After normalization to genotype-specific baseline values, the relative magnitude of the response to moderate exposure (100 rESWs) was largely comparable between wild-type worms and *rpy-1*-KOs. In contrast, clearer genotype-dependent differences emerged at the higher exposure level (300 rESWs), where several locomotor parameters showed stronger deviations in the mutant animals. Taken together, these observations indicate that RPY-1 modulates the locomotor response when the neuromuscular system is challenged by stronger mechanical perturbation induced by rESWs. Rather than indicating a uniform increase in vulnerability, the findings suggest that this effect is parameter-dependent and, in the recovery experiment, also phase-dependent.

This interpretation is consistent with the well-established role of rapsyn as a central organizer of postsynaptic receptor clustering. Rapsyn links AChRs to the cytoskeletal scaffold of the synapse and is essential for the formation and stabilization of the high-density AChR clusters required for efficient neuromuscular transmission [18,19]. The reduced locomotor performance observed in *rpy-1*-KOs therefore strongly suggests that RPY-1-dependent receptor clustering also contributes to synaptic function in *C. elegans*. At the same time, the present results indicate that the absence of RPY-1 did not abolish locomotor function. Even in the absence of RPY-1, animals retained substantial locomotor activity and tolerated moderate levels of mechanical stimulation induced by rESWs. This finding suggests that postsynaptic AChR organization is not maintained by rapsyn (or RPY-1) alone but rather by a cooperative molecular network.

In vertebrate NMJs, the postsynaptic specialization consists of a highly organized multiprotein complex that includes the agrin–MuSK–LRP4 signaling pathway as well as several cytoskeletal and anchoring proteins that stabilize AChR clusters [20,35,36,37,38,39]. Within this network, rapsyn functions as a key scaffolding molecule that promotes receptor clustering and links the receptors to the underlying cytoskeleton. The existence of such a multiprotein scaffold provides a plausible explanation for the present observations. When rapsyn (or RPY-1 in *C. elegans*) is absent, other molecular interactions may still maintain partial receptor organization, allowing the NMJ to function under baseline conditions (albeit with reduced efficiency). However, such a partially compromised system may respond differently to mechanical perturbation, such that stronger stimuli—such as exposure to 300 rESWs—produce additional functional alterations.

This mechanistic interpretation is compatible with previous work in rats, demonstrating that rESWs can induce structural alterations of the postsynaptic region of the NMJ, including changes in the morphology of AChR clusters [11,12,13]. The present findings extend these observations by suggesting that molecules involved in postsynaptic receptor organization may influence the behavioral response to such perturbations. However, the present study did not directly assess AChR clustering, the structural integrity of the postsynaptic apparatus, or neuromuscular transmission. Likewise, RPY-1::GFP imaging was exploratory and qualitative and therefore does not allow definitive conclusions regarding subtle changes in protein abundance, localization, or nanoscale organization. Accordingly, present data do not directly establish altered postsynaptic scaffold stability, but rather identify loss of RPY-1 as a factor that modifies the acute locomotor response to rESW exposure. A recent preprint reported that *C. elegans* worms exposed not only to blast-generated shock waves but also to rESWs generated with the same rESWT device used here, although with a slightly different exposure protocol, showed immediate locomotor impairment followed by recovery during the subsequent day, whereas longer-term deterioration emerged later and was associated with progressive neuronal dysfunction [26]. Because this work has not yet undergone peer review, these findings should be considered provisional. Nevertheless, the behavioral patterns observed in the present study are consistent with the early phase of the response described in [26]: locomotor impairment occurred immediately after rESW exposure and was followed by substantial recovery during the first hours. Because the present study specifically focused on short-term effects within a recovery period of up to three hours, later time points were not examined. It therefore remains possible that, under the experimental conditions used here, longer-term neuronal effects may also occur in *C. elegans*. Accordingly, the present findings should be interpreted as describing the early phase of the response to rESWs rather than the full temporal spectrum of possible biological effects.

When the results of the present study are considered in a broader physiological and clinical context, an apparent paradox emerges. Experimental and clinical observations suggest that rESWs can influence neuromuscular transmission at the level of the NMJ. In clinical practice, rESWT performed with the same device used in the present study has been successfully applied in the treatment of acute muscle injuries in elite athletes without causing detectable muscle weakness and, when integrated into multimodal rehabilitation programs, has been associated with shortened recovery times and improved functional outcomes [40,41]. Specifically, a retrospective analysis of elite football players demonstrated substantially reduced lay-off times when rESWT was incorporated into treatment protocols for acute muscle injuries [40], and a randomized controlled trial of athletes with acute hamstring muscle complex injuries showed faster return-to-sport after rESWT compared with sham treatment [41]. These observations indicate that rESWT can promote functional recovery of injured skeletal muscle without compromising muscle performance.

This situation appears paradoxical when compared with the therapeutic effects of rESWT in spasticity, where clinical improvement is thought to involve partial weakening of neuromuscular transmission. The resolution of this apparent contradiction likely lies in the fact that the NMJ itself is altered under conditions of spasticity. Increasing evidence indicates that central motor lesions can induce structural remodeling of the NMJ, including enlarged endplates, altered AChR distribution and increased polyaxonal innervation [42]. Such changes suggest that the NMJ in spastic muscle operates under different structural and functional constraints than in healthy muscle. Consequently, interventions that influence the structure or stability of the NMJ may have fundamentally different functional consequences depending on the physiological state of the neuromuscular system.

Within this conceptual framework, therapeutic interventions that influence the structure or stability of the NMJ (as in the case of rESWT as suggested by prior work [11,12,13], and potentially also by the results of the present study) may potentially modulate spastic motor patterns. The present findings suggest that the molecular architecture of the postsynaptic receptor-clustering machinery represents one factor that may determine how the NMJ responds to mechanical perturbations such as rESW exposure. In this context, the organization of the postsynaptic scaffold may influence whether mechanical stimulation produces only transient functional effects or leads to more pronounced alterations of synaptic organization.

The present study also highlights the complementary strengths and limitations of different experimental models. The *C. elegans* system allows genetic manipulation of individual molecular components such as the *rapsyn* homolog *rpy-1* while maintaining viable organisms, thereby providing a powerful approach for dissecting the molecular mechanisms that stabilize postsynaptic receptor clusters. In contrast, mammalian models are required to study the complex central mechanisms that underlie spasticity. However, these systems also have limitations. For example, *rapsyn* knockout mice are not viable because functional neuromuscular junctions cannot form in the absence of rapsyn [17], which makes it difficult to investigate the specific contribution of rapsyn-dependent mechanisms in mammalian models. At the same time, the present behavioral data do not allow one to distinguish conclusively between specific NMJ-related mechanisms and broader physiological susceptibility; rescue experiments would be required to address this question more directly.

The declared conflict of interest should also be considered when interpreting the findings. One author (C.S.) has served as a consultant to and received research funding from the manufacturer of the rESWT device used in this study. The corresponding author (T.H.) has no conflicts of interest to declare. The behavioral analyses were performed using a fully automated tracking system, and the disclosure is provided here in the interest of transparency.

Taken together, the present findings support the view that RPY-1 contributes to the modulation of locomotor responses to mechanical perturbation induced by rESWs. The data are compatible with the role of postsynaptic receptor organization in this context, but they do not directly establish the underlying cellular mechanism. Integrating molecular insights obtained from genetically tractable model organisms with physiological studies in mammalian systems may therefore provide an important strategy for understanding how mechanical interventions such as rESWT influence neuromuscular transmission and motor function in conditions such as spasticity.

## 5. Conclusions

Radial extracorporeal shock waves induced pronounced alterations in locomotor behavior in *C. elegans*, affecting multiple parameters of movement including speed, trajectory body-wave dynamics. These effects were most prominent immediately after exposure and were largely reversible within the first hours. Comparison between wild-type worms and *rpy-1* knockout mutants showed that loss of the rapsyn homolog RPY-1 modified the behavioral response to rESW exposure. These effects were dose-dependent and varied across locomotor parameters, indicating that the influence of RPY-1 on the response to mechanical perturbation is parameter-specific. The findings of this study are consistent with the idea that components involved in neuromuscular signaling contribute to the behavioral effects of rESW exposure. However, the present data do not directly assess AChR clustering, neuromuscular junction structure, or neuromuscular transmission.

## Figures and Tables

**Table 1:** Summary of the statistical analysis (*p* values) of the relative data shown in Figure 3. Abbreviations: PS, peristaltic speed; TL, track length; WL, wavelength; R, reversals; MA, mean amplitude; WT, wild-type; *rpy-1*(ok145) knockout strain NM1581, *rpy-1*-KO.

	PS	TL	WL	R	MA
Normality testing (Shapiro–Wilk test)					
Group					
WT/0	<0.001	<0.001	0.003	<0.001	<0.001
WT/100	<0.001	<0.001	<0.001	<0.001	<0.001
WT/300	<0.001	<0.001	<0.001	<0.001	<0.001
*rpy-1*-KO/0	<0.001	<0.001	<0.001	<0.001	<0.001
*rpy-1*-KO/100	<0.001	<0.001	<0.001	<0.001	<0.001
*rpy-1*-KO/300	<0.001	<0.001	<0.001	<0.001	<0.001
Non-parametric two-factor analysis (Scheirer-Ray-Hare test)					
Genotype	0.554	0.127	<0.001	<0.001	<0.001
Exposure	<0.001	<0.001	<0.001	<0.001	<0.001
Genotype × Exposure	0.001	0.001	<0.001	0.005	<0.001
Post hoc pairwise comparisons (Dunn-type comparisons; Mann–Whitney tests with Holm correction					
-Within wildtype					
WT/0 vs. WT/100	<0.001	<0.001	0.003	<0.001	0.026
WT/0 vs. WT/300	<0.001	<0.001	0.003	<0.001	<0.001
WT/100 vs. WT/300	<0.001	<0.001	0.836	0.001	<0.001
-Within *rpy-1*-KO					
*rpy-1*-KO/0 vs. *rpy-1*-KO/100	<0.001	<0.001	<0.001	<0.001	<0.001
*rpy-1*-KO/0 vs. *rpy-1*-KO/300	<0.001	<0.001	<0.001	<0.001	<0.001
*rpy-1*-KO/100 vs. *rpy-1*-KO/300	0.004	0.004	0.003	0.097	<0.001
-Between genotypes (same exposure)					
WT/0 vs. *rpy-1*-KO/0	0.779	0.618	0.548	0.740	0.458
WT/100 vs. *rpy-1*-KO/100	0.779	0.865	0.006	0.128	<0.001
WT/300 vs. *rpy-1*-KO/300	<0.001	<0.001	<0.001	<0.001	<0.001

**Table 2:** Summary of the statistical analysis (*p* values) of the relative data shown in Figure 4. Abbreviations: PS, peristaltic speed; TL, track length; WL, wavelength; R, reversals; MA, mean amplitude; WT, wild-type; *rpy-1*(ok145) knockout strain NM1581, *rpy-1*-KO.

	PS	TL	WL	R	MA
Normality testing (Shapiro–Wilk test)					
Group					
WT/0/0	0.059	0.129	<0.001	<0.001	<0.001
WT/300/0	<0.001	<0.001	0.099	0.041	<0.001
WT/300/30	<0.001	<0.001	0.003	<0.001	<0.001
WT/300/180	<0.001	<0.001	<0.001	<0.001	<0.001
*rpy-1*-KO/0/0	0.028	0.175	<0.001	<0.001	<0.001
*rpy-1*-KO/300/0	<0.001	0.001	0.002	0.001	<0.001
*rpy-1*-KO/300/30	0.007	0.001	0.007	<0.001	<0.001
*rpy-1*-KO/300/180	<0.001	<0.001	<0.001	<0.001	<0.001
Non-parametric two-factor analysis (Scheirer-Ray-Hare test)					
Genotype	<0.001	<0.001	0.727	0.531	0.305
Exposure	<0.001	<0.001	0.005	<0.001	<0.001
Recovery	1.000	1.000	1.000	1.000	1.000
Genotype × Exposure	0.008	0.010	0.725	0.621	0.767
Genotype × Recovery	1.000	1.000	1.000	1.000	1.000
Exposure × Recovery	<0.001	<0.001	<0.001	<0.001	<0.001
Genotype × Exposure × Recovery	<0.001	<0.001	0.465	<0.001	0.681
Post hoc pairwise comparisons (Dunn-type comparisons; Mann–Whitney tests with Holm correction					
-Baseline comparison					
WT/0/0 vs. *rpy-1*-KO/0/0	0.869	0.813	0.401	0.939	0.649
-Immediate effects after exposure (0 min)					
WT/0/0 vs. WT/300/0	<0.001	<0.001	<0.001	<0.001	<0.001
*rpy-1*-KO/0/0 vs. *rpy-1*-KO/300/0	<0.001	<0.001	0.043	<0.001	<0.001
WT/300/0 vs. *rpy-1*-KO/300/0	0.318	0.249	0.683	<0.001	0.757
-Effects at 30 min after exposure					
WT/300/0 vs. WT/300/30	<0.001	<0.001	0.028	<0.001	<0.001
*rpy-1*-KO/300/0 vs. *rpy-1*-KO/300/30	<0.001	<0.001	0.757	<0.001	<0.001
WT/300/30 vs. *rpy-1*-KO/300/30	0.015	0.049	0.952	0.216	0.809
-Effects at 180 min after exposure					
WT/300/0 vs. WT/300/180	<0.001	<0.001	<0.001	<0.001	<0.001
*rpy-1*-KO/300/0 vs. *rpy-1*-KO/300/180	<0.001	<0.001	0.757	<0.001	<0.001
WT/300/180 vs. *rpy-1*-KO/300/180	<0.001	<0.001	0.952	0.031	0.892

## Data Availability

The data that support the findings of this study are available on request from the corresponding author.

## Supplementary Materials

The following supporting information can be downloaded at: https://www.mdpi.com/article/10.3390/biomedicines14050960/s1. File S1: Python code for statistical analyses.

## Abbreviations

The following abbreviations are used in this manuscript:

rESWT	Radial extracorporeal shock wave therapy
rESWs	Radial extracorporeal shock waves
NMJ	Neuromuscular junction
AChR	Acetylcholine receptor
*C. elegans*	*Caenorhabditis elegans*
GFP	green fluorescent protein
