# Supplementary material for: Rapsyn Homolog RPY-1 Modulates Locomotor Responses of Caenorhabditis elegans to Radial Extracorporeal Shock Waves
Source: Biomedicines. 2026 Apr 22;14(5):960. doi: 10.3390/biomedicines14050960 (PMC13203884; doi:10.3390/biomedicines14050960)
Supplement: Supplementary file 1 [file biomedicines-14-00960-s001.zip › biomedicines-4247203-supplementary.pdf]

Rapsyn Homolog rpy-1 Modulates Locomotor Responses of Caenorhabditis elegans to Radial Extracorporeal Shock Waves

by T. Hochstrasser, L. Kaub und C. Schmitz

Supplementary File S1

Python code for statistical analyses

Table of contents

1. Python code
2. First experiment
3. Second experiment
4. List of installed Python packages

---

1. Python code

1.1 Imports

In [1]:
```
import itertools
from pathlib import Path
import os

import numpy as np
import pandas as pd
from scipy.stats import shapiro, rankdata, mannwhitneyu, chi2
import statsmodels.api as sm
import statsmodels.formula.api as smf
from statsmodels.stats.multitest import multipletests
```

1.2 Functions

1.2.1 Data input

In [2]:
```
def format_p(p):
    """Format p-values for reporting."""
    if pd.isna(p):
        return "NA"
    if p < 0.001:
        return "<0.001"
    return f"{p:.6g}"


def interp_p(p, alpha=0.05):
    return "significant" if p < alpha else "not significant"


def load_excel(path, sheet_name=0):
    df = pd.read_excel(path, sheet_name=sheet_name)
    df.columns = [str(c).strip() for c in df.columns]
    return df


def load_wide_excel(path, sheet_name=0):
    """
    Load a wide-format Excel file where each column is "genotype / exposure" and each row contains values.
    Returns a long-format DataFrame with columns: genotype, exposure, value
    """
    df_raw = pd.read_excel(path, sheet_name=sheet_name)
    df_raw.columns = [str(c).strip() for c in df_raw.columns]

    long_data = []
    for col in df_raw.columns:
        # split column name into genotype and exposure
        try:
            genotype, exposure = [x.strip() for x in col.split("/")]
        except ValueError:
            raise ValueError(f"Column name '{col}' is not in 'genotype / exposure' format")
        for val in df_raw[col].dropna():
```

```
            long_data.append({
                "genotype": genotype,
                "exposure": exposure,
                "value": float(val)
            })

    df_long = pd.DataFrame(long_data)
    return df_long


def prepare_columns(df, value_col, genotype_col, exposure_col, recovery_col):
    out = df.copy()

    for col in [value_col, genotype_col, exposure_col] + ([recovery_col] if recovery_col else []):
        if col not in out.columns:
            raise ValueError(f"Missing required column: {col}")

    out = out[[value_col, genotype_col, exposure_col] + ([recovery_col] if recovery_col else [])].copy()
    out = out.dropna()

    out[value_col] = pd.to_numeric(out[value_col], errors="coerce")
    out = out.dropna(subset=[value_col])

    out[genotype_col] = out[genotype_col].astype(str).str.strip()
    out[exposure_col] = out[exposure_col].astype(str).str.strip()
    if recovery_col:
        out[recovery_col] = out[recovery_col].astype(str).str.strip()

    return out


def prepare_wide_columns(df):
    """
    Ensure that the long-format dataframe has correct types for the first series:
    columns: 'genotype', 'exposure', 'value'
    """
    df = df[["genotype", "exposure", "value"]].copy()
    df["genotype"] = df["genotype"].astype(str).str.strip()
    df["exposure"] = df["exposure"].astype(str).str.strip()
    df["value"] = pd.to_numeric(df["value"], errors="coerce")
    df = df.dropna(subset=["value"])
    return df


def load_wide_excel_3factor(path, sheet_name=0, value_name="value"):
    """
    Load a wide-format Excel file where each column is "genotype / exposure / recovery"
    and each row contains measurements. Converts to long-format DataFrame with
    columns: genotype, exposure, recovery, <value_name>
    """
    df_raw = pd.read_excel(path, sheet_name=sheet_name)
    df_raw.columns = [str(c).strip() for c in df_raw.columns]

    long_data = []
    for col in df_raw.columns:
        try:
            genotype, exposure, recovery = [x.strip() for x in col.split("/")]
        except ValueError:
            raise ValueError(f"Column name '{col}' is not in 'genotype / exposure / recovery' format")
        for val in df_raw[col].dropna():
            long_data.append({
                "genotype": genotype,
                "exposure": exposure,
                "recovery": recovery,
                value_name: float(val)
            })

    df_long = pd.DataFrame(long_data)
    return df_long


def prepare_wide_columns_3factor(df, value_name="value"):
    """
    Ensure correct types for 3-factor long-format dataframe:
    'genotype', 'exposure', 'recovery', <value_name>
    """
    df = df[["genotype", "exposure", "recovery", value_name]].copy()
    df["genotype"] = df["genotype"].astype(str).str.strip()
    df["exposure"] = df["exposure"].astype(str).str.strip()
    df["recovery"] = df["recovery"].astype(str).str.strip()
    df[value_name] = pd.to_numeric(df[value_name], errors="coerce")
    df = df.dropna(subset=[value_name])
    return df
```

1.2.2 Statistical tests

In [3]:
```python
def shapiro_by_groups(df, value_col, group_cols):
    rows = []
    grouped = df.groupby(group_cols, dropna=False)

    for keys, sub in grouped:
        vals = sub[value_col].dropna().to_numpy()
        if len(vals) < 3:
            p = np.nan
            interpretation = "too few values"
        else:
            _, p = shapiro(vals)
            interpretation = "approximately normal" if p >= 0.05 else "not normally distributed"

        if not isinstance(keys, tuple):
            keys = (keys,)

        row = {col: val for col, val in zip(group_cols, keys)}
        row["n"] = len(vals)
        row["p_value"] = p
        row["p_value_formatted"] = format_p(p) if pd.notna(p) else "NA"
        row["interpretation"] = interpretation
        rows.append(row)

    return pd.DataFrame(rows)


def scheirer_ray_hare(df, value_col, factor_a, factor_b):
    """
    Scheirer-Ray-Hare (2-factor)
    This implementation follows the standard SRH logic:
    rank response globally, compute sums of squares on ranks,
    divide by mean square error, and test H ~ chi-square(df).
    """
    d = df[[value_col, factor_a, factor_b]].dropna().copy()
    d["_rank"] = rankdata(d[value_col], method="average")

    N = len(d)
    grand_mean = d["_rank"].mean()

    levels_a = d[factor_a].unique()
    levels_b = d[factor_b].unique()

    # Cell means and sizes
    cell = d.groupby([factor_a, factor_b])["_rank"].agg(["mean", "count"]).reset_index()
    a_tab = d.groupby(factor_a)["_rank"].agg(["mean", "count"]).reset_index()
    b_tab = d.groupby(factor_b)["_rank"].agg(["mean", "count"]).reset_index()

    # Total SS on ranks
    SS_total = ((d["_rank"] - grand_mean) ** 2).sum()

    # Main effects
    SS_a = ((a_tab["count"] * (a_tab["mean"] - grand_mean) ** 2)).sum()
    SS_b = ((b_tab["count"] * (b_tab["mean"] - grand_mean) ** 2)).sum()

    # Cell SS
    SS_cells = ((cell["count"] * (cell["mean"] - grand_mean) ** 2)).sum()

    # Interaction and residual
    SS_ab = SS_cells - SS_a - SS_b
    SS_error = SS_total - SS_cells

    df_a = len(levels_a) - 1
    df_b = len(levels_b) - 1
    df_ab = df_a * df_b
    df_error = N - len(levels_a) * len(levels_b)

    MS_error = SS_error / df_error

    H_a = SS_a / MS_error
    H_b = SS_b / MS_error
    H_ab = SS_ab / MS_error

    p_a = 1 - chi2.cdf(H_a, df_a)
    p_b = 1 - chi2.cdf(H_b, df_b)
    p_ab = 1 - chi2.cdf(H_ab, df_ab)

    result = pd.DataFrame([
        {"Factor": factor_a, "df": df_a, "H": H_a, "p_value": p_a, "interpretation": interp_p(p_a)},
        {"Factor": factor_b, "df": df_b, "H": H_b, "p_value": p_b, "interpretation": interp_p(p_b)},
        {"Factor": f"{factor_a} × {factor_b}", "df": df_ab, "H": H_ab, "p_value": p_ab, "interpretation": interp_p(p_ab)},
    ])
    result["p_value_formatted"] = result["p_value"].map(format_p)
    return result


def rank_based_anova_3way(df, value_col, genotype_col, exposure_col, recovery_col):
    """
```

```python
    Rank-based 3-factor ANOVA: rank the response, then fit a full factorial ANOVA on the ranks.
    """
    d = df[[value_col, genotype_col, exposure_col, recovery_col]].dropna().copy()
    d["_rank"] = rankdata(d[value_col], method="average")

    formula = (
        f"_rank ~ C({genotype_col}) * C({exposure_col}) * C({recovery_col})"
    )
    model = smf.ols(formula, data=d).fit()
    anova = sm.stats.anova_lm(model, typ=2).reset_index()
    anova = anova.rename(columns={"index": "Factor", "PR(>F)": "p_value", "df": "df", "F": "F_stat"})

    # clean factor labels
    def clean_factor(name):
        return (
            name.replace(f"C({genotype_col})", genotype_col)
                .replace(f"C({exposure_col})", exposure_col)
                .replace(f"C({recovery_col})", recovery_col)
                .replace(":", " × ")
        )

    anova["Factor"] = anova["Factor"].map(clean_factor)
    anova["interpretation"] = anova["p_value"].apply(
        lambda p: interp_p(p) if pd.notna(p) else "NA"
    )
    anova["p_value_formatted"] = anova["p_value"].apply(
        lambda p: format_p(p) if pd.notna(p) else "NA"
    )

    return anova[["Factor", "df", "F_stat", "p_value", "p_value_formatted", "interpretation"]]


def pairwise_mannwhitney(df, value_col, group_cols, comparisons, alternative="two-sided"):
    """
    Pairwise Mann-Whitney + Holm
    group_cols example:
      [genotype_col, exposure_col]
      [genotype_col, exposure_col, recovery_col]

    comparisons:
      list of ((group1_level1, group1_level2, ...), (group2_level1, group2_level2, ...))
    """
    d = df.copy()

    grouped = {
        key if isinstance(key, tuple) else (key,): sub[value_col].dropna().to_numpy()
        for key, sub in d.groupby(group_cols, dropna=False)
    }

    if comparisons is None:
        keys = sorted(grouped.keys())
        comparisons = list(itertools.combinations(keys, 2))

    raw_ps = []
    rows = []

    for g1, g2 in comparisons:
        x = grouped[g1]
        y = grouped[g2]

        stat, p = mannwhitneyu(x, y, alternative=alternative)

        rows.append({
            "group1": g1,
            "group2": g2,
            "U": stat,
            "p_raw": p
        })
        raw_ps.append(p)

    # Holm correction
    reject, p_adj, _, _ = multipletests(raw_ps, method="holm")

    for row, padj, rej in zip(rows, p_adj, reject):
        row["p_adjusted"] = padj
        row["p_adjusted_formatted"] = format_p(padj)
        row["significant"] = bool(rej)

    out = pd.DataFrame(rows)
    return out


def label_group(group_tuple):
    return " / ".join(map(str, group_tuple))


def comparisons_first_series(genotypes, exposures):
```

```python
    out = {}

    within = []
    for g in genotypes:
        for e1, e2 in itertools.combinations(exposures, 2):
            within.append(((g, e1), (g, e2)))
    out["within_genotype"] = within

    between = []
    for e in exposures:
        if len(genotypes) != 2:
            raise ValueError("between-genotype same-exposure comparisons assume exactly 2 genotypes")
        between.append(((genotypes[0], e), (genotypes[1], e)))
    out["between_genotypes_same_exposure"] = between

    return out


def comparisons_second_series(genotypes, exposure_control, exposure_treated, recovery_levels):
    if len(genotypes) != 2:
        raise ValueError("This helper assumes exactly 2 genotypes")

    g1, g2 = genotypes

    out = {}

    # baseline genotype comparison
    out["baseline_genotype"] = [
        ((g1, exposure_control, "0"), (g2, exposure_control, "0"))
    ]

    # immediate effects
    out["immediate_within_genotype"] = [
        ((g1, exposure_control, "0"), (g1, exposure_treated, "0")),
        ((g2, exposure_control, "0"), (g2, exposure_treated, "0")),
    ]

    out["immediate_between_genotype"] = [
        ((g1, exposure_treated, "0"), (g2, exposure_treated, "0"))
    ]

    # recovery within genotype
    rec_within = []
    for g in genotypes:
        if "30" in recovery_levels:
            rec_within.append(((g, exposure_treated, "0"), (g, exposure_treated, "30")))
        if "180" in recovery_levels:
            rec_within.append(((g, exposure_treated, "0"), (g, exposure_treated, "180")))
        if "30" in recovery_levels and "180" in recovery_levels:
            rec_within.append(((g, exposure_treated, "30"), (g, exposure_treated, "180")))
    out["recovery_within_genotype"] = rec_within

    # genotype comparison during recovery
    rec_between = []
    for r in recovery_levels:
        if r != "0":
            rec_between.append(((g1, exposure_treated, r), (g2, exposure_treated, r)))
    out["recovery_between_genotype"] = rec_between

    # baseline vs recovery within genotype
    base_vs_rec = []
    for g in genotypes:
        for r in recovery_levels:
            if r != "0":
                base_vs_rec.append(((g, exposure_control, "0"), (g, exposure_treated, r)))
    out["baseline_vs_recovery"] = base_vs_rec

    return out
```

1.2.3 Functions to run analysis

In [4]:

```python
def analyze_first_series(excel_path, value_col="value", sheet_name=0):
    """
    Analyze first experimental series from a wide-format Excel file
    where columns are 'genotype / exposure' and rows are measurements.

    Parameters
    ----------
    excel_path : str | Path
        Path to Excel file
    value_col : str
        Name of the value column (will be created in reshaping)
    sheet_name : int | str
        Excel sheet name or index

    Returns
```

```
    -------
    dict with keys: normality, srh, posthoc
    """

    # 1. Load wide Excel and convert to long format
    df = load_wide_excel(excel_path, sheet_name=sheet_name)
    df = prepare_wide_columns(df)

    # 2. Normality testing per group
    normality = shapiro_by_groups(df, value_col, ["genotype", "exposure"])

    # 3. Scheirer-Ray-Hare test
    srh = scheirer_ray_hare(df, value_col, "genotype", "exposure")

    # 4. Post-hoc pairwise comparisons (9 comparisons)
    genotypes = sorted(df["genotype"].astype(str).unique())
    exposures = sorted(df["exposure"].astype(str).unique(), key=lambda x: float(x) if x.replace(".", "", 1).isdigit() else x
    comp_sets = comparisons_first_series(genotypes, exposures)

    posthoc_results = {}
    for title, comps in comp_sets.items():
        res = pairwise_mannwhitney(
            df=df,
            value_col=value_col,
            group_cols=["genotype", "exposure"],
            comparisons=comps
        )
        res["Comparison"] = res.apply(
            lambda r: f"{label_group(r['group1'])} vs {label_group(r['group2'])}",
            axis=1
        )
        # reorder columns so Comparison is right after group2
        cols = ["group1", "group2", "Comparison"] + [c for c in res.columns if c not in ["group1", "group2", "Comparison"]]
        res = res[cols]
        posthoc_results[title] = res

    # 5. Return structured results
    return {
        "normality": normality,
        "srh": srh,
        "posthoc": posthoc_results
    }
```

In [5]:
```
def analyze_second_series(excel_path, value_name="value", control_exposure= "0", treated_exposure="300", sheet_name=0):
    """
    Analyze second experimental series from a wide-format Excel file
    where columns are 'genotype / exposure / recovery'.
    Sets the measurement column to <value_name>.
    """

    # 1. Load and reshape
    df = load_wide_excel_3factor(excel_path, sheet_name=sheet_name, value_name=value_name)
    df = prepare_wide_columns_3factor(df, value_name=value_name)

    # 2. Normality testing
    normality = shapiro_by_groups(df, value_name, ["genotype", "exposure", "recovery"])

    # 3. Rank-based three-way ANOVA
    anova3 = rank_based_anova_3way(df, value_name, "genotype", "exposure", "recovery")

    # 4. Post-hoc pairwise comparisons
    genotypes = sorted(df["genotype"].astype(str).unique())
    recoveries = sorted(df["recovery"].astype(str).unique(), key=lambda x: float(x) if x.replace(".", "", 1).isdigit() else

    comp_sets = comparisons_second_series(
        genotypes=genotypes,
        exposure_control=str(control_exposure),
        exposure_treated=str(treated_exposure),
        recovery_levels=recoveries
    )

    posthoc_results = {}
    for title, comps in comp_sets.items():
        res = pairwise_mannwhitney(
            df=df,
            value_col=value_name,
            group_cols=["genotype", "exposure", "recovery"],
            comparisons=comps
        )

        res["Comparison"] = res.apply(lambda r: f"{label_group(r['group1'])} vs {label_group(r['group2'])}", axis=1)
        cols = ["group1", "group2", "Comparison"] + [c for c in res.columns if c not in ["group1", "group2", "Comparison"]]
        res = res[cols]

        posthoc_results[title] = res

    return {
```

```
        "normality": normality,
        "anova3": anova3,
        "posthoc": posthoc_results
    }
```

---

2. First experiment

2.1 Peristaltic speed

In [6]:
```
speed = analyze_first_series(excel_path="data v2/1-1-rel Absolute peristaltic speed.xlsx")
```

In [7]:
```
speed["normality"]
```

Out[7]:

	genotype	exposure	n	p_value	p_value_formatted	interpretation
**0**	N2	0	313	9.036415e-04	<0.001	not normally distributed
**1**	N2	100	400	1.237886e-20	<0.001	not normally distributed
**2**	N2	300	345	1.906243e-23	<0.001	not normally distributed
**3**	rpy-1	0	386	5.055383e-10	<0.001	not normally distributed
**4**	rpy-1	100	430	1.996268e-28	<0.001	not normally distributed
**5**	rpy-1	300	423	3.104703e-30	<0.001	not normally distributed

In [8]:
```
speed["srh"]
```

Out[8]:

	Factor	df	H	p_value	interpretation	p_value_formatted
**0**	genotype	1	0.349126	0.554608	not significant	0.554608
**1**	exposure	2	1302.465907	0.000000	significant	<0.001
**2**	genotype × exposure	2	13.255441	0.001323	significant	0.00132318

In [9]:
```
speed["posthoc"]["within_genotype"]
```

Out[9]:

	group1	group2	Comparison	U	p_raw	p_adjusted	p_adjusted_formatted	significant
**0**	(N2, 0)	(N2, 100)	N2 / 0 vs N2 / 100	103964.5	6.999098e-52	2.099730e-51	<0.001	True
**1**	(N2, 0)	(N2, 300)	N2 / 0 vs N2 / 300	99146.5	9.454431e-77	4.727216e-76	<0.001	True
**2**	(N2, 100)	(N2, 300)	N2 / 100 vs N2 / 300	85873.5	8.377239e-09	1.675448e-08	<0.001	True
**3**	(rpy-1, 0)	(rpy-1, 100)	rpy-1 / 0 vs rpy-1 / 100	138613.0	1.700775e-61	6.803099e-61	<0.001	True
**4**	(rpy-1, 0)	(rpy-1, 300)	rpy-1 / 0 vs rpy-1 / 300	145565.0	1.265283e-82	7.591699e-82	<0.001	True
**5**	(rpy-1, 100)	(rpy-1, 300)	rpy-1 / 100 vs rpy-1 / 300	101377.5	3.737404e-03	3.737404e-03	0.0037374	True

In [10]:
```
speed["posthoc"]["between_genotypes_same_exposure"]
```

Out[10]:

	group1	group2	Comparison	U	p_raw	p_adjusted	p_adjusted_formatted	significant
**0**	(N2, 0)	(rpy-1, 0)	N2 / 0 vs rpy-1 / 0	62549.0	0.420293	0.779756	0.779756	False
**1**	(N2, 100)	(rpy-1, 100)	N2 / 100 vs rpy-1 / 100	88968.0	0.389878	0.779756	0.779756	False
**2**	(N2, 300)	(rpy-1, 300)	N2 / 300 vs rpy-1 / 300	59920.0	0.000020	0.000060	<0.001	True

2.2 Track length

In [11]:
```
length = analyze_first_series(excel_path="data v2/1-2-rel Track Length.xlsx")
```

In [12]:
```
length["normality"]
```

Out[12]:

	genotype	exposure	n	p_value	p_value_formatted	interpretation
**0**	N2	0	313	8.603921e-07	<0.001	not normally distributed
**1**	N2	100	400	5.673375e-21	<0.001	not normally distributed
**2**	N2	300	345	6.491664e-24	<0.001	not normally distributed
**3**	rpy-1	0	386	2.371341e-13	<0.001	not normally distributed
**4**	rpy-1	100	430	7.498975e-29	<0.001	not normally distributed
**5**	rpy-1	300	423	6.566700e-29	<0.001	not normally distributed

In [13]:
```
length["srh"]
```

Out[13]:

	Factor	df	H	p_value	interpretation	p_value_formatted
**0**	genotype	1	2.329256	0.126963	not significant	0.126963
**1**	exposure	2	1226.484006	0.000000	significant	<0.001
**2**	genotype × exposure	2	13.662640	0.001079	significant	0.00107943

In [14]:
```
length["posthoc"]["within_genotype"]
```

Out[14]:

	group1	group2	Comparison	U	p_raw	p_adjusted	p_adjusted_formatted	significant
**0**	(N2, 0)	(N2, 100)	N2 / 0 vs N2 / 100	102091.0	1.908202e-47	5.724607e-47	<0.001	True
**1**	(N2, 0)	(N2, 300)	N2 / 0 vs N2 / 300	97939.5	8.420866e-73	4.210433e-72	<0.001	True
**2**	(N2, 100)	(N2, 300)	N2 / 100 vs N2 / 300	85791.0	9.895155e-09	1.979031e-08	<0.001	True
**3**	(rpy-1, 0)	(rpy-1, 100)	rpy-1 / 0 vs rpy-1 / 100	137592.0	2.518991e-59	1.007596e-58	<0.001	True
**4**	(rpy-1, 0)	(rpy-1, 300)	rpy-1 / 0 vs rpy-1 / 300	145335.0	4.810060e-82	2.886036e-81	<0.001	True
**5**	(rpy-1, 100)	(rpy-1, 300)	rpy-1 / 100 vs rpy-1 / 300	101395.0	3.679863e-03	3.679863e-03	0.00367986	True

In [15]:
```
length["posthoc"]["between_genotypes_same_exposure"]
```

Out[15]:

	group1	group2	Comparison	U	p_raw	p_adjusted	p_adjusted_formatted	significant
**0**	(N2, 0)	(rpy-1, 0)	N2 / 0 vs rpy-1 / 0	63111.0	3.088640e-01	6.177280e-01	0.617728	False
**1**	(N2, 100)	(rpy-1, 100)	N2 / 100 vs rpy-1 / 100	86586.0	8.652857e-01	8.652857e-01	0.865286	False
**2**	(N2, 300)	(rpy-1, 300)	N2 / 300 vs rpy-1 / 300	57322.0	3.122242e-07	9.366727e-07	<0.001	True

2.3 Wavelength

In [16]:
```
wavelength = analyze_first_series(excel_path="data v2/1-3-rel Wavelength.xlsx")
```

In [17]:
```
wavelength["normality"]
```

Out[17]:

	genotype	exposure	n	p_value	p_value_formatted	interpretation
**0**	N2	0	312	2.522028e-03	0.00252203	not normally distributed
**1**	N2	100	400	7.568581e-10	<0.001	not normally distributed
**2**	N2	300	345	5.446177e-05	<0.001	not normally distributed
**3**	rpy-1	0	386	6.669504e-06	<0.001	not normally distributed
**4**	rpy-1	100	430	2.314097e-07	<0.001	not normally distributed
**5**	rpy-1	300	423	3.882412e-06	<0.001	not normally distributed

In [18]:
```
wavelength["srh"]
```

Out[18]:

	Factor	df	H	p_value	interpretation	p_value_formatted
**0**	genotype	1	24.294906	8.265735e-07	significant	<0.001
**1**	exposure	2	105.871297	0.000000e+00	significant	<0.001
**2**	genotype × exposure	2	20.011973	4.512894e-05	significant	<0.001

In [19]:
```
wavelength["posthoc"]["within_genotype"]
```

Out[19]:

	group1	group2	Comparison	U	p_raw	p_adjusted	p_adjusted_formatted	significant
**0**	(N2, 0)	(N2, 100)	N2 / 0 vs N2 / 100	53483.0	1.058830e-03	3.176490e-03	0.00317649	True
**1**	(N2, 0)	(N2, 300)	N2 / 0 vs N2 / 300	45993.5	1.276121e-03	3.176490e-03	0.00317649	True
**2**	(N2, 100)	(N2, 300)	N2 / 100 vs N2 / 300	68391.5	8.355570e-01	8.355570e-01	0.835557	False
**3**	(rpy-1, 0)	(rpy-1, 100)	rpy-1 / 0 vs rpy-1 / 100	60384.0	1.760868e-11	8.804339e-11	<0.001	True
**4**	(rpy-1, 0)	(rpy-1, 300)	rpy-1 / 0 vs rpy-1 / 300	48677.0	3.123804e-23	1.874282e-22	<0.001	True
**5**	(rpy-1, 100)	(rpy-1, 300)	rpy-1 / 100 vs rpy-1 / 300	78654.5	6.356418e-04	2.542567e-03	0.00254257	True

In [20]:

```
wavelength["posthoc"]["between_genotypes_same_exposure"]
```

Out[20]:

	group1	group2	Comparison	U	p_raw	p_adjusted	p_adjusted_formatted	significant
**0**	(N2, 0)	(rpy-1, 0)	N2 / 0 vs rpy-1 / 0	61807.0	5.481714e-01	5.481714e-01	0.548171	False
**1**	(N2, 100)	(rpy-1, 100)	N2 / 100 vs rpy-1 / 100	75794.0	3.105874e-03	6.211748e-03	0.00621175	True
**2**	(N2, 300)	(rpy-1, 300)	N2 / 300 vs rpy-1 / 300	55987.0	2.816775e-08	8.450324e-08	<0.001	True

2.4 Reversals

In [21]:

```
reversals = analyze_first_series(excel_path="data v2/1-4-rel Reversals.xlsx")
```

In [22]:

```
reversals["normality"]
```

Out[22]:

	genotype	exposure	n	p_value	p_value_formatted	interpretation
**0**	N2	0	313	5.288635e-05	<0.001	not normally distributed
**1**	N2	100	400	2.585612e-04	<0.001	not normally distributed
**2**	N2	300	345	1.133073e-06	<0.001	not normally distributed
**3**	rpy-1	0	386	7.593558e-07	<0.001	not normally distributed
**4**	rpy-1	100	430	2.955282e-10	<0.001	not normally distributed
**5**	rpy-1	300	423	1.511307e-10	<0.001	not normally distributed

In [23]:

```
reversals["srh"]
```

Out[23]:

	Factor	df	H	p_value	interpretation	p_value_formatted
**0**	genotype	1	10.961782	0.000930	significant	<0.001
**1**	exposure	2	220.210398	0.000000	significant	<0.001
**2**	genotype × exposure	2	10.451305	0.005377	significant	0.00537685

In [24]:

```
reversals["posthoc"]["within_genotype"]
```

Out[24]:

	group1	group2	Comparison	U	p_raw	p_adjusted	p_adjusted_formatted	significant
**0**	(N2, 0)	(N2, 100)	N2 / 0 vs N2 / 100	41230.0	4.557245e-15	1.822898e-14	<0.001	True
**1**	(N2, 0)	(N2, 300)	N2 / 0 vs N2 / 300	28567.5	1.411965e-25	8.471790e-25	<0.001	True
**2**	(N2, 100)	(N2, 300)	N2 / 100 vs N2 / 300	58878.0	5.319120e-04	1.063824e-03	0.00106382	True
**3**	(rpy-1, 0)	(rpy-1, 100)	rpy-1 / 0 vs rpy-1 / 100	60011.0	7.785769e-12	2.335731e-11	<0.001	True
**4**	(rpy-1, 0)	(rpy-1, 300)	rpy-1 / 0 vs rpy-1 / 300	52808.0	3.586831e-18	1.793415e-17	<0.001	True
**5**	(rpy-1, 100)	(rpy-1, 300)	rpy-1 / 100 vs rpy-1 / 300	84981.0	9.677456e-02	9.677456e-02	0.0967746	False

In [25]:

```
reversals["posthoc"]["between_genotypes_same_exposure"]
```

Out[25]:

	group1	group2	Comparison	U	p_raw	p_adjusted	p_adjusted_formatted	significant
**0**	(N2, 0)	(rpy-1, 0)	N2 / 0 vs rpy-1 / 0	59527.0	7.397970e-01	7.397970e-01	0.739797	False
**1**	(N2, 100)	(rpy-1, 100)	N2 / 100 vs rpy-1 / 100	92385.0	6.418334e-02	1.283667e-01	0.128367	False
**2**	(N2, 300)	(rpy-1, 300)	N2 / 300 vs rpy-1 / 300	89411.0	7.445877e-08	2.233763e-07	<0.001	True

2.5 Amplitude

In [26]:
```
amplitude = analyze_first_series(excel_path="data v2/1-5-rel Mean amplitude.xlsx")
```

In [27]:
```
amplitude["normality"]
```

Out[27]:

	genotype	exposure	n	p_value	p_value_formatted	interpretation
**0**	N2	0	313	4.329860e-04	<0.001	not normally distributed
**1**	N2	100	400	3.655101e-07	<0.001	not normally distributed
**2**	N2	300	345	1.096038e-08	<0.001	not normally distributed
**3**	rpy-1	0	386	1.927923e-11	<0.001	not normally distributed
**4**	rpy-1	100	430	9.958800e-11	<0.001	not normally distributed
**5**	rpy-1	300	423	2.271703e-17	<0.001	not normally distributed

In [28]:
```
amplitude["srh"]
```

Out[28]:

	Factor	df	H	p_value	interpretation	p_value_formatted
**0**	genotype	1	77.417149	0.000000e+00	significant	<0.001
**1**	exposure	2	273.329725	0.000000e+00	significant	<0.001
**2**	genotype × exposure	2	29.458503	4.010212e-07	significant	<0.001

In [29]:
```
amplitude["posthoc"]["within_genotype"]
```

Out[29]:

	group1	group2	Comparison	U	p_raw	p_adjusted	p_adjusted_formatted	significant
**0**	(N2, 0)	(N2, 100)	N2 / 0 vs N2 / 100	68690.0	2.567381e-02	2.567381e-02	0.0256738	True
**1**	(N2, 0)	(N2, 300)	N2 / 0 vs N2 / 300	68145.0	6.193596e-09	1.858079e-08	<0.001	True
**2**	(N2, 100)	(N2, 300)	N2 / 100 vs N2 / 300	80593.5	7.557483e-05	1.511497e-04	<0.001	True
**3**	(rpy-1, 0)	(rpy-1, 100)	rpy-1 / 0 vs rpy-1 / 100	110092.5	7.493823e-16	3.746912e-15	<0.001	True
**4**	(rpy-1, 0)	(rpy-1, 300)	rpy-1 / 0 vs rpy-1 / 300	131988.5	5.925654e-52	3.555393e-51	<0.001	True
**5**	(rpy-1, 100)	(rpy-1, 300)	rpy-1 / 100 vs rpy-1 / 300	118926.5	7.417577e-15	2.967031e-14	<0.001	True

In [30]:
```
amplitude["posthoc"]["between_genotypes_same_exposure"]
```

Out[30]:

	group1	group2	Comparison	U	p_raw	p_adjusted	p_adjusted_formatted	significant
**0**	(N2, 0)	(rpy-1, 0)	N2 / 0 vs rpy-1 / 0	62382.0	4.574775e-01	4.574775e-01	0.457477	False
**1**	(N2, 100)	(rpy-1, 100)	N2 / 100 vs rpy-1 / 100	104546.0	7.718459e-08	1.543692e-07	<0.001	True
**2**	(N2, 300)	(rpy-1, 300)	N2 / 300 vs rpy-1 / 300	94264.0	3.312334e-12	9.937002e-12	<0.001	True

SAVE RESULTS

In [31]:
```
parameters = ["speed", "length", "wavelength", "reversals", "amplitude"]

out_folder = "results"
os.makedirs(out_folder, exist_ok=True)

for value_name in parameters:
    results = globals()[value_name]

    out_xlsx = os.path.join(out_folder, f"results_{value_name}_first.xlsx")

    with pd.ExcelWriter(out_xlsx, engine="openpyxl") as writer:
        results["normality"].to_excel(writer, sheet_name="normality", index=False)
        results["srh"].to_excel(writer, sheet_name="scheirer", index=True)
        for comp_name, df in results["posthoc"].items():
            sheet_name = comp_name[:31]
            df.to_excel(writer, sheet_name=sheet_name, index=False)
```

---

3. Second experiment

3.1 Peristaltic speed

In [32]:
```
speed2 = analyze_second_series(
    excel_path="data v2/2-1-rel Absolute peristaltic speed.xlsx",
```

```
    value_name="Peristaltic Speed",
    control_exposure="0", treated_exposure="300")
```

In [33]:
```
speed2["normality"]
```

Out[33]:

	genotype	exposure	recovery	n	p_value	p_value_formatted	interpretation
**0**	WT	0	0	137	5.866925e-02	0.0586692	approximately normal
**1**	WT	300	0	107	3.488674e-18	<0.001	not normally distributed
**2**	WT	300	180	131	5.911902e-05	<0.001	not normally distributed
**3**	WT	300	30	104	4.184396e-04	<0.001	not normally distributed
**4**	rpy-1	0	0	95	2.766529e-02	0.0276653	not normally distributed
**5**	rpy-1	300	0	67	3.026825e-04	<0.001	not normally distributed
**6**	rpy-1	300	180	85	2.032676e-05	<0.001	not normally distributed
**7**	rpy-1	300	30	89	6.484507e-03	0.00648451	not normally distributed

In [34]:
```
speed2["anova3"]
```

Out[34]:

	Factor	df	F_stat	p_value	p_value_formatted	interpretation
**0**	genotype	1.0	2.274745e+01	2.192072e-06	<0.001	significant
**1**	exposure	1.0	2.545498e+02	5.167712e-50	<0.001	significant
**2**	recovery	2.0	-1.717479e-13	1.000000e+00	1	not significant
**3**	genotype × exposure	1.0	7.104013e+00	7.844876e-03	0.00784488	significant
**4**	genotype × recovery	2.0	3.878368e-13	1.000000e+00	1	not significant
**5**	exposure × recovery	2.0	2.004357e+02	2.128689e-71	<0.001	significant
**6**	genotype × exposure × recovery	2.0	8.443837e+00	2.348179e-04	<0.001	significant
**7**	Residual	807.0	NaN	NaN	NA	NA

In [35]:
```
speed2["posthoc"]["baseline_genotype"]
```

Out[35]:

	group1	group2	Comparison	U	p_raw	p_adjusted	p_adjusted_formatted	significant
**0**	(WT, 0, 0)	(rpy-1, 0, 0)	WT / 0 / 0 vs rpy-1 / 0 / 0	6424.0	0.868859	0.868859	0.868859	False

In [36]:
```
speed2["posthoc"]["immediate_within_genotype"]
```

Out[36]:

	group1	group2	Comparison	U	p_raw	p_adjusted	p_adjusted_formatted	significant
**0**	(WT, 0, 0)	(WT, 300, 0)	WT / 0 / 0 vs WT / 300 / 0	14256.5	9.717872e-37	1.943574e-36	<0.001	True
**1**	(rpy-1, 0, 0)	(rpy-1, 300, 0)	rpy-1 / 0 / 0 vs rpy-1 / 300 / 0	6339.5	6.961359e-27	6.961359e-27	<0.001	True

In [37]:
```
speed2["posthoc"]["immediate_between_genotype"]
```

Out[37]:

	group1	group2	Comparison	U	p_raw	p_adjusted	p_adjusted_formatted	significant
**0**	(WT, 300, 0)	(rpy-1, 300, 0)	WT / 300 / 0 vs rpy-1 / 300 / 0	3261.0	0.317813	0.317813	0.317813	False

In [38]:
```
speed2["posthoc"]["recovery_within_genotype"]
```

Out[38]:

	group1	group2	Comparison	U	p_raw	p_adjusted	p_adjusted_formatted	significant
**0**	(WT, 300, 0)	(WT, 300, 30)	WT / 300 / 0 vs WT / 300 / 30	1460.5	2.168009e-20	8.672038e-20	<0.001	True
**1**	(WT, 300, 0)	(WT, 300, 180)	WT / 300 / 0 vs WT / 300 / 180	1352.5	9.774192e-27	5.864515e-26	<0.001	True
**2**	(WT, 300, 30)	(WT, 300, 180)	WT / 300 / 30 vs WT / 300 / 180	6413.0	4.413838e-01	4.413838e-01	0.441384	False
**3**	(rpy-1, 300, 0)	(rpy-1, 300, 30)	rpy-1 / 300 / 0 vs rpy-1 / 300 / 30	352.5	4.928501e-21	2.464251e-20	<0.001	True
**4**	(rpy-1, 300, 0)	(rpy-1, 300, 180)	rpy-1 / 300 / 0 vs rpy-1 / 300 / 180	387.0	6.893700e-20	2.068110e-19	<0.001	True
**5**	(rpy-1, 300, 30)	(rpy-1, 300, 180)	rpy-1 / 300 / 30 vs rpy-1 / 300 / 180	2673.0	8.412128e-04	1.682426e-03	0.00168243	True

In [39]:
```
speed2["posthoc"]["recovery_between_genotype"]
```

Out[39]:

	group1	group2	Comparison	U	p_raw	p_adjusted	p_adjusted_formatted	significant
0	(WT, 300, 30)	(rpy-1, 300, 30)	WT / 300 / 30 vs rpy-1 / 300 / 30	3690.0	0.015370	0.015370	0.0153703	True
1	(WT, 300, 180)	(rpy-1, 300, 180)	WT / 300 / 180 vs rpy-1 / 300 / 180	3566.0	0.000008	0.000016	<0.001	True

In [40]:
```
speed2["posthoc"]["baseline_vs_recovery"]
```

Out[40]:

	group1	group2	Comparison	U	p_raw	p_adjusted	p_adjusted_formatted	significant
0	(WT, 0, 0)	(WT, 300, 30)	WT / 0 / 0 vs WT / 300 / 30	10893.5	2.046946e-12	8.187784e-12	<0.001	True
1	(WT, 0, 0)	(WT, 300, 180)	WT / 0 / 0 vs WT / 300 / 180	13200.5	2.674576e-11	8.023729e-11	<0.001	True
2	(rpy-1, 0, 0)	(rpy-1, 300, 30)	rpy-1 / 0 / 0 vs rpy-1 / 300 / 30	5629.5	1.036544e-04	2.073089e-04	<0.001	True
3	(rpy-1, 0, 0)	(rpy-1, 300, 180)	rpy-1 / 0 / 0 vs rpy-1 / 300 / 180	3555.0	1.672454e-01	1.672454e-01	0.167245	False

3.2 Track length

In [41]:
```
length2 = analyze_second_series(
    excel_path="data v2/2-2-rel Track length.xlsx",
    value_name="Track Length",
    control_exposure="0", treated_exposure="300")
```

In [42]:
```
length2["normality"]
```

Out[42]:

	genotype	exposure	recovery	n	p_value	p_value_formatted	interpretation
0	WT	0	0	137	1.292399e-01	0.12924	approximately normal
1	WT	300	0	107	4.106152e-18	<0.001	not normally distributed
2	WT	300	180	131	3.428070e-05	<0.001	not normally distributed
3	WT	300	30	104	3.359138e-04	<0.001	not normally distributed
4	rpy-1	0	0	95	1.751924e-01	0.175192	approximately normal
5	rpy-1	300	0	67	1.173656e-03	0.00117366	not normally distributed
6	rpy-1	300	180	85	7.631475e-05	<0.001	not normally distributed
7	rpy-1	300	30	89	1.499727e-03	0.00149973	not normally distributed

In [43]:
```
length2["anova3"]
```

Out[43]:

	Factor	df	F_stat	p_value	p_value_formatted	interpretation
0	genotype	1.0	2.157359e+01	3.974507e-06	<0.001	significant
1	exposure	1.0	2.476023e+02	7.387259e-49	<0.001	significant
2	recovery	2.0	-1.330758e-13	1.000000e+00	1	not significant
3	genotype × exposure	1.0	6.608673e+00	1.032651e-02	0.0103265	significant
4	genotype × recovery	2.0	3.673911e-13	1.000000e+00	1	not significant
5	exposure × recovery	2.0	2.006474e+02	1.848023e-71	<0.001	significant
6	genotype × exposure × recovery	2.0	8.724793e+00	1.783427e-04	<0.001	significant
7	Residual	807.0	NaN	NaN	NA	NA

In [44]:
```
length2["posthoc"]["baseline_genotype"]
```

Out[44]:

	group1	group2	Comparison	U	p_raw	p_adjusted	p_adjusted_formatted	significant
0	(WT, 0, 0)	(rpy-1, 0, 0)	WT / 0 / 0 vs rpy-1 / 0 / 0	6388.0	0.812873	0.812873	0.812873	False

In [45]:
```
length2["posthoc"]["baseline_genotype"]
```

Out[45]:

	group1	group2	Comparison	U	p_raw	p_adjusted	p_adjusted_formatted	significant
0	(WT, 0, 0)	(rpy-1, 0, 0)	WT / 0 / 0 vs rpy-1 / 0 / 0	6388.0	0.812873	0.812873	0.812873	False

In [46]:
```
length2["posthoc"]["immediate_within_genotype"]
```

Out[46]:

	group1	group2	Comparison	U	p_raw	p_adjusted	p_adjusted_formatted	significant
**0**	(WT, 0, 0)	(WT, 300, 0)	WT / 0 / 0 vs WT / 300 / 0	14223.0	2.116778e-36	4.233556e-36	<0.001	True
**1**	(rpy-1, 0, 0)	(rpy-1, 300, 0)	rpy-1 / 0 / 0 vs rpy-1 / 300 / 0	6345.0	5.690716e-27	5.690716e-27	<0.001	True

In [47]:
```
length2["posthoc"]["immediate_between_genotype"]
```

Out[47]:

	group1	group2	Comparison	U	p_raw	p_adjusted	p_adjusted_formatted	significant
**0**	(WT, 300, 0)	(rpy-1, 300, 0)	WT / 300 / 0 vs rpy-1 / 300 / 0	3211.0	0.248669	0.248669	0.248669	False

In [48]:
```
length2["posthoc"]["recovery_within_genotype"]
```

Out[48]:

	group1	group2	Comparison	U	p_raw	p_adjusted	p_adjusted_formatted	significant
**0**	(WT, 300, 0)	(WT, 300, 30)	WT / 300 / 0 vs WT / 300 / 30	1470.0	2.649335e-20	7.948004e-20	<0.001	True
**1**	(WT, 300, 0)	(WT, 300, 180)	WT / 300 / 0 vs WT / 300 / 180	1378.0	1.644155e-26	9.864932e-26	<0.001	True
**2**	(WT, 300, 30)	(WT, 300, 180)	WT / 300 / 30 vs WT / 300 / 180	6519.0	5.720209e-01	5.720209e-01	0.572021	False
**3**	(rpy-1, 300, 0)	(rpy-1, 300, 30)	rpy-1 / 300 / 0 vs rpy-1 / 300 / 30	328.0	2.133554e-21	1.066777e-20	<0.001	True
**4**	(rpy-1, 300, 0)	(rpy-1, 300, 180)	rpy-1 / 300 / 0 vs rpy-1 / 300 / 180	348.0	1.793932e-20	7.175729e-20	<0.001	True
**5**	(rpy-1, 300, 30)	(rpy-1, 300, 180)	rpy-1 / 300 / 30 vs rpy-1 / 300 / 180	2649.0	6.469653e-04	1.293931e-03	0.00129393	True

In [49]:
```
length2["posthoc"]["recovery_between_genotype"]
```

Out[49]:

	group1	group2	Comparison	U	p_raw	p_adjusted	p_adjusted_formatted	significant
**0**	(WT, 300, 30)	(rpy-1, 300, 30)	WT / 300 / 30 vs rpy-1 / 300 / 30	3866.0	0.049004	0.049004	0.049004	True
**1**	(WT, 300, 180)	(rpy-1, 300, 180)	WT / 300 / 180 vs rpy-1 / 300 / 180	3459.0	0.000003	0.000005	<0.001	True

In [50]:
```
length2["posthoc"]["baseline_vs_recovery"]
```

Out[50]:

	group1	group2	Comparison	U	p_raw	p_adjusted	p_adjusted_formatted	significant
**0**	(WT, 0, 0)	(WT, 300, 30)	WT / 0 / 0 vs WT / 300 / 30	10757.0	1.230401e-11	4.921605e-11	<0.001	True
**1**	(WT, 0, 0)	(WT, 300, 180)	WT / 0 / 0 vs WT / 300 / 180	13142.0	4.990337e-11	1.497101e-10	<0.001	True
**2**	(rpy-1, 0, 0)	(rpy-1, 300, 30)	rpy-1 / 0 / 0 vs rpy-1 / 300 / 30	5645.0	8.680094e-05	1.736019e-04	<0.001	True
**3**	(rpy-1, 0, 0)	(rpy-1, 300, 180)	rpy-1 / 0 / 0 vs rpy-1 / 300 / 180	3596.0	2.063642e-01	2.063642e-01	0.206364	False

3.3 Wavelength

In [51]:
```
wavelength2 = analyze_second_series(
    excel_path="data v2/2-3-rel Wavelength.xlsx",
    value_name="Wavelength",
    control_exposure="0", treated_exposure="300")
```

In [52]:
```
wavelength2["normality"]
```

Out[52]:

	genotype	exposure	recovery	n	p_value	p_value_formatted	interpretation
**0**	WT	0	0	137	4.239839e-06	<0.001	not normally distributed
**1**	WT	300	0	107	9.882573e-02	0.0988257	approximately normal
**2**	WT	300	180	131	9.407016e-06	<0.001	not normally distributed
**3**	WT	300	30	104	2.758023e-03	0.00275802	not normally distributed
**4**	rpy-1	0	0	95	5.383757e-06	<0.001	not normally distributed
**5**	rpy-1	300	0	67	2.374729e-03	0.00237473	not normally distributed
**6**	rpy-1	300	180	85	2.361444e-11	<0.001	not normally distributed
**7**	rpy-1	300	30	89	6.937589e-03	0.00693759	not normally distributed

In [53]:
```
wavelength2["anova3"]
```

Out[53]:

	Factor	df	F_stat	p_value	p_value_formatted	interpretation
**0**	genotype	1.0	1.219873e-01	0.726980	0.72698	not significant
**1**	exposure	1.0	7.867248e+00	0.005155	0.00515484	significant
**2**	recovery	2.0	-3.659701e-14	1.000000	1	not significant
**3**	genotype × exposure	1.0	1.241782e-01	0.724638	0.724638	not significant
**4**	genotype × recovery	2.0	-1.563428e-14	1.000000	1	not significant
**5**	exposure × recovery	2.0	6.976764e+00	0.000991	<0.001	significant
**6**	genotype × exposure × recovery	2.0	7.670276e-01	0.464730	0.46473	not significant
**7**	Residual	807.0	NaN	NaN	NA	NA

In [54]:
```
wavelength2["posthoc"]["baseline_genotype"]
```

Out[54]:

	group1	group2	Comparison	U	p_raw	p_adjusted	p_adjusted_formatted	significant
**0**	(WT, 0, 0)	(rpy-1, 0, 0)	WT / 0 / 0 vs rpy-1 / 0 / 0	6930.0	0.401208	0.401208	0.401208	False

In [55]:
```
wavelength2["posthoc"]["immediate_within_genotype"]
```

Out[55]:

	group1	group2	Comparison	U	p_raw	p_adjusted	p_adjusted_formatted	significant
**0**	(WT, 0, 0)	(WT, 300, 0)	WT / 0 / 0 vs WT / 300 / 0	5415.0	0.000468	0.000935	<0.001	True
**1**	(rpy-1, 0, 0)	(rpy-1, 300, 0)	rpy-1 / 0 / 0 vs rpy-1 / 300 / 0	2587.0	0.043016	0.043016	0.043016	True

In [56]:
```
wavelength2["posthoc"]["immediate_between_genotype"]
```

Out[56]:

	group1	group2	Comparison	U	p_raw	p_adjusted	p_adjusted_formatted	significant
**0**	(WT, 300, 0)	(rpy-1, 300, 0)	WT / 300 / 0 vs rpy-1 / 300 / 0	3717.0	0.683097	0.683097	0.683097	False

In [57]:
```
wavelength2["posthoc"]["recovery_within_genotype"]
```

Out[57]:

	group1	group2	Comparison	U	p_raw	p_adjusted	p_adjusted_formatted	significant
**0**	(WT, 300, 0)	(WT, 300, 30)	WT / 300 / 0 vs WT / 300 / 30	6794.0	0.005555	0.027775	0.0277748	True
**1**	(WT, 300, 0)	(WT, 300, 180)	WT / 300 / 0 vs WT / 300 / 180	9067.5	0.000098	0.000587	<0.001	True
**2**	(WT, 300, 30)	(WT, 300, 180)	WT / 300 / 30 vs WT / 300 / 180	7492.0	0.189277	0.757109	0.757109	False
**3**	(rpy-1, 300, 0)	(rpy-1, 300, 30)	rpy-1 / 300 / 0 vs rpy-1 / 300 / 30	3316.5	0.231080	0.757109	0.757109	False
**4**	(rpy-1, 300, 0)	(rpy-1, 300, 180)	rpy-1 / 300 / 0 vs rpy-1 / 300 / 180	3173.0	0.227781	0.757109	0.757109	False
**5**	(rpy-1, 300, 30)	(rpy-1, 300, 180)	rpy-1 / 300 / 30 vs rpy-1 / 300 / 180	3830.0	0.887473	0.887473	0.887473	False

In [58]:
```
wavelength2["posthoc"]["recovery_between_genotype"]
```

Out[58]:

	group1	group2	Comparison	U	p_raw	p_adjusted	p_adjusted_formatted	significant
**0**	(WT, 300, 30)	(rpy-1, 300, 30)	WT / 300 / 30 vs rpy-1 / 300 / 30	4736.0	0.781090	0.951537	0.951537	False
**1**	(WT, 300, 180)	(rpy-1, 300, 180)	WT / 300 / 180 vs rpy-1 / 300 / 180	5247.0	0.475768	0.951537	0.951537	False

In [59]:
```
wavelength2["posthoc"]["baseline_vs_recovery"]
```

Out[59]:

	group1	group2	Comparison	U	p_raw	p_adjusted	p_adjusted_formatted	significant
**0**	(WT, 0, 0)	(WT, 300, 30)	WT / 0 / 0 vs WT / 300 / 30	6277.5	0.114507	0.458030	0.45803	False
**1**	(WT, 0, 0)	(WT, 300, 180)	WT / 0 / 0 vs WT / 300 / 180	8842.0	0.836374	0.836374	0.836374	False
**2**	(rpy-1, 0, 0)	(rpy-1, 300, 30)	rpy-1 / 0 / 0 vs rpy-1 / 300 / 30	3775.0	0.210588	0.458030	0.45803	False
**3**	(rpy-1, 0, 0)	(rpy-1, 300, 180)	rpy-1 / 0 / 0 vs rpy-1 / 300 / 180	3517.0	0.136226	0.458030	0.45803	False

3.4 Reversals

In [60]:
```
reversals2 = analyze_second_series(
    excel_path="data v2/2-4-rel Reversals.xlsx",
    value_name="Reversals",
    control_exposure="0", treated_exposure="300")
```

In [61]:
```
reversals2["normality"]
```

Out[61]:

	genotype	exposure	recovery	n	p_value	p_value_formatted	interpretation
**0**	WT	0	0	137	7.160046e-14	<0.001	not normally distributed
**1**	WT	300	0	107	4.084488e-02	0.0408449	not normally distributed
**2**	WT	300	180	131	2.238512e-10	<0.001	not normally distributed
**3**	WT	300	30	104	4.363358e-11	<0.001	not normally distributed
**4**	rpy-1	0	0	95	2.692920e-12	<0.001	not normally distributed
**5**	rpy-1	300	0	67	1.074575e-03	0.00107458	not normally distributed
**6**	rpy-1	300	180	85	1.019944e-12	<0.001	not normally distributed
**7**	rpy-1	300	30	89	4.640760e-12	<0.001	not normally distributed

In [62]:
```
reversals2["anova3"]
```

Out[62]:

	Factor	df	F_stat	p_value	p_value_formatted	interpretation
**0**	genotype	1.0	3.930549e-01	5.308753e-01	0.530875	not significant
**1**	exposure	1.0	6.957657e+01	3.164300e-16	<0.001	significant
**2**	recovery	2.0	-1.376828e-14	1.000000e+00	1	not significant
**3**	genotype × exposure	1.0	2.446825e-01	6.209791e-01	0.620979	not significant
**4**	genotype × recovery	2.0	1.164201e-13	1.000000e+00	1	not significant
**5**	exposure × recovery	2.0	1.441021e+02	3.070458e-54	<0.001	significant
**6**	genotype × exposure × recovery	2.0	9.046050e+00	1.302391e-04	<0.001	significant
**7**	Residual	807.0	NaN	NaN	NA	NA

In [63]:
```
reversals2["posthoc"]["baseline_genotype"]
```

Out[63]:

	group1	group2	Comparison	U	p_raw	p_adjusted	p_adjusted_formatted	significant
**0**	(WT, 0, 0)	(rpy-1, 0, 0)	WT / 0 / 0 vs rpy-1 / 0 / 0	6545.0	0.939476	0.939476	0.939476	False

In [64]:
```
reversals2["posthoc"]["immediate_within_genotype"]
```

Out[64]:

	group1	group2	Comparison	U	p_raw	p_adjusted	p_adjusted_formatted	significant
**0**	(WT, 0, 0)	(WT, 300, 0)	WT / 0 / 0 vs WT / 300 / 0	1509.5	1.044125e-26	2.088251e-26	<0.001	True
**1**	(rpy-1, 0, 0)	(rpy-1, 300, 0)	rpy-1 / 0 / 0 vs rpy-1 / 300 / 0	366.0	3.852259e-22	3.852259e-22	<0.001	True

In [65]:
```
reversals2["posthoc"]["immediate_between_genotype"]
```

Out[65]:

	group1	group2	Comparison	U	p_raw	p_adjusted	p_adjusted_formatted	significant
**0**	(WT, 300, 0)	(rpy-1, 300, 0)	WT / 300 / 0 vs rpy-1 / 300 / 0	1838.5	6.561892e-08	6.561892e-08	<0.001	True

In [66]:
```
reversals2["posthoc"]["recovery_within_genotype"]
```

Out[66]:

	group1	group2	Comparison	U	p_raw	p_adjusted	p_adjusted_formatted	significant
**0**	(WT, 300, 0)	(WT, 300, 30)	WT / 300 / 0 vs WT / 300 / 30	9263.5	5.704705e-17	2.852352e-16	<0.001	True
**1**	(WT, 300, 0)	(WT, 300, 180)	WT / 300 / 0 vs WT / 300 / 180	10976.5	5.029902e-14	1.508971e-13	<0.001	True
**2**	(WT, 300, 30)	(WT, 300, 180)	WT / 300 / 30 vs WT / 300 / 180	5969.5	9.829475e-02	1.965895e-01	0.196589	False
**3**	(rpy-1, 300, 0)	(rpy-1, 300, 30)	rpy-1 / 300 / 0 vs rpy-1 / 300 / 30	5543.0	1.796507e-20	1.077904e-19	<0.001	True
**4**	(rpy-1, 300, 0)	(rpy-1, 300, 180)	rpy-1 / 300 / 0 vs rpy-1 / 300 / 180	5038.5	1.917941e-16	7.671765e-16	<0.001	True
**5**	(rpy-1, 300, 30)	(rpy-1, 300, 180)	rpy-1 / 300 / 30 vs rpy-1 / 300 / 180	3835.0	8.680748e-01	8.680748e-01	0.868075	False

In [67]:
```
reversals2["posthoc"]["recovery_between_genotype"]
```

Out[67]:

	group1	group2	Comparison	U	p_raw	p_adjusted	p_adjusted_formatted	significant
**0**	(WT, 300, 30)	(rpy-1, 300, 30)	WT / 300 / 30 vs rpy-1 / 300 / 30	5092.0	0.215500	0.215500	0.2155	False
**1**	(WT, 300, 180)	(rpy-1, 300, 180)	WT / 300 / 180 vs rpy-1 / 300 / 180	6628.0	0.015383	0.030767	0.0307669	True

In [68]:
```
reversals2["posthoc"]["baseline_vs_recovery"]
```

Out[68]:

	group1	group2	Comparison	U	p_raw	p_adjusted	p_adjusted_formatted	significant
0	(WT, 0, 0)	(WT, 300, 30)	WT / 0 / 0 vs WT / 300 / 30	6443.5	0.193843	0.58153	0.58153	False
1	(WT, 0, 0)	(WT, 300, 180)	WT / 0 / 0 vs WT / 300 / 180	6938.5	0.001087	0.00435	0.00434951	True
2	(rpy-1, 0, 0)	(rpy-1, 300, 30)	rpy-1 / 0 / 0 vs rpy-1 / 300 / 30	4343.5	0.736492	1.00000	1	False
3	(rpy-1, 0, 0)	(rpy-1, 300, 180)	rpy-1 / 0 / 0 vs rpy-1 / 300 / 180	4221.0	0.580455	1.00000	1	False

3.5 Amplitude

In [69]:
```
amplitude2 = analyze_second_series(
    excel_path="data v2/2-5-rel Mean amplitude.xlsx",
    value_name="Mean Amplitude",
    control_exposure="0", treated_exposure="300")
```

In [70]:
```
amplitude2["normality"]
```

Out[70]:

	genotype	exposure	recovery	n	p_value	p_value_formatted	interpretation
0	WT	0	0	137	4.801208e-07	<0.001	not normally distributed
1	WT	300	0	107	2.224101e-09	<0.001	not normally distributed
2	WT	300	180	131	1.689765e-10	<0.001	not normally distributed
3	WT	300	30	104	7.008593e-05	<0.001	not normally distributed
4	rpy-1	0	0	95	7.603853e-05	<0.001	not normally distributed
5	rpy-1	300	0	67	1.173628e-08	<0.001	not normally distributed
6	rpy-1	300	180	85	7.207508e-11	<0.001	not normally distributed
7	rpy-1	300	30	89	2.579807e-04	<0.001	not normally distributed

In [71]:
```
amplitude2["anova3"]
```

Out[71]:

	Factor	df	F_stat	p_value	p_value_formatted	interpretation
0	genotype	1.0	1.052416e+00	3.052587e-01	0.305259	not significant
1	exposure	1.0	4.407041e+01	5.812263e-11	<0.001	significant
2	recovery	2.0	-3.512020e-14	1.000000e+00	1	not significant
3	genotype × exposure	1.0	8.816582e-02	7.665984e-01	0.766598	not significant
4	genotype × recovery	2.0	-5.672606e-15	1.000000e+00	1	not significant
5	exposure × recovery	2.0	7.561417e+01	7.956584e-31	<0.001	significant
6	genotype × exposure × recovery	2.0	3.846364e-01	6.808228e-01	0.680823	not significant
7	Residual	807.0	NaN	NaN	NA	NA

In [72]:
```
amplitude2["posthoc"]["baseline_genotype"]
```

Out[72]:

	group1	group2	Comparison	U	p_raw	p_adjusted	p_adjusted_formatted	significant
0	(WT, 0, 0)	(rpy-1, 0, 0)	WT / 0 / 0 vs rpy-1 / 0 / 0	6737.0	0.648721	0.648721	0.648721	False

In [73]:
```
amplitude2["posthoc"]["immediate_within_genotype"]
```

Out[73]:

	group1	group2	Comparison	U	p_raw	p_adjusted	p_adjusted_formatted	significant
0	(WT, 0, 0)	(WT, 300, 0)	WT / 0 / 0 vs WT / 300 / 0	11933.0	3.966208e-17	7.932416e-17	<0.001	True
1	(rpy-1, 0, 0)	(rpy-1, 300, 0)	rpy-1 / 0 / 0 vs rpy-1 / 300 / 0	5549.0	8.511471e-16	8.511471e-16	<0.001	True

In [74]:
```
amplitude2["posthoc"]["immediate_between_genotype"]
```

Out[74]:

	group1	group2	Comparison	U	p_raw	p_adjusted	p_adjusted_formatted	significant
0	(WT, 300, 0)	(rpy-1, 300, 0)	WT / 300 / 0 vs rpy-1 / 300 / 0	3685.0	0.757113	0.757113	0.757113	False

In [75]:
```
amplitude2["posthoc"]["recovery_within_genotype"]
```

Out[75]:

	group1	group2	Comparison	U	p_raw	p_adjusted	p_adjusted_formatted	significant
**0**	(WT, 300, 0)	(WT, 300, 30)	WT / 300 / 0 vs WT / 300 / 30	2613.5	2.866052e-11	9.618344e-11	<0.001	True
**1**	(WT, 300, 0)	(WT, 300, 180)	WT / 300 / 0 vs WT / 300 / 180	3479.0	2.404586e-11	9.618344e-11	<0.001	True
**2**	(WT, 300, 30)	(WT, 300, 180)	WT / 300 / 30 vs WT / 300 / 180	7828.0	4.978146e-02	9.956291e-02	0.0995629	False
**3**	(rpy-1, 300, 0)	(rpy-1, 300, 30)	rpy-1 / 300 / 0 vs rpy-1 / 300 / 30	1082.0	1.054738e-11	5.273689e-11	<0.001	True
**4**	(rpy-1, 300, 0)	(rpy-1, 300, 180)	rpy-1 / 300 / 0 vs rpy-1 / 300 / 180	975.5	3.777593e-12	2.266556e-11	<0.001	True
**5**	(rpy-1, 300, 30)	(rpy-1, 300, 180)	rpy-1 / 300 / 30 vs rpy-1 / 300 / 180	4122.5	3.067174e-01	3.067174e-01	0.306717	False

In [76]:
```
amplitude2["posthoc"]["recovery_between_genotype"]
```

Out[76]:

	group1	group2	Comparison	U	p_raw	p_adjusted	p_adjusted_formatted	significant
**0**	(WT, 300, 30)	(rpy-1, 300, 30)	WT / 300 / 30 vs rpy-1 / 300 / 30	4951.0	0.404452	0.808904	0.808904	False
**1**	(WT, 300, 180)	(rpy-1, 300, 180)	WT / 300 / 180 vs rpy-1 / 300 / 180	5506.0	0.891869	0.891869	0.891869	False

In [77]:
```
amplitude2["posthoc"]["baseline_vs_recovery"]
```

Out[77]:

	group1	group2	Comparison	U	p_raw	p_adjusted	p_adjusted_formatted	significant
**0**	(WT, 0, 0)	(WT, 300, 30)	WT / 0 / 0 vs WT / 300 / 30	6909.0	0.689039	0.994778	0.994778	False
**1**	(WT, 0, 0)	(WT, 300, 180)	WT / 0 / 0 vs WT / 300 / 180	10545.5	0.013226	0.052906	0.0529058	False
**2**	(rpy-1, 0, 0)	(rpy-1, 300, 30)	rpy-1 / 0 / 0 vs rpy-1 / 300 / 30	4473.0	0.497389	0.994778	0.994778	False
**3**	(rpy-1, 0, 0)	(rpy-1, 300, 180)	rpy-1 / 0 / 0 vs rpy-1 / 300 / 180	4673.5	0.068615	0.205846	0.205846	False

SAVE RESULTS

In [78]:
```
parameters = ["speed2", "length2", "wavelength2", "reversals2", "amplitude2"]

out_folder = "results"
os.makedirs(out_folder, exist_ok=True)

for value_name in parameters:
    results = globals()[value_name]

    out_xlsx = os.path.join(out_folder, f"results_{value_name}_second.xlsx")

    with pd.ExcelWriter(out_xlsx, engine="openpyxl") as writer:
        results["normality"].to_excel(writer, sheet_name="normality", index=False)
        results["anova3"].to_excel(writer, sheet_name="3-way-ANOVA", index=True)
        for comp_name, df in results["posthoc"].items():
            sheet_name = comp_name[:31]
            df.to_excel(writer, sheet_name=sheet_name, index=False)
```

4. List of installed Python packages

In [3]:
```
conda list
```

```
# packages in environment at C:\Users\Leon\miniconda3\envs\analyze:
#
# Name                    Version                   Build  Channel
aiofiles                  22.1.0           py39haa95532_0
aiosqlite                 0.18.0           py39haa95532_0
anyio                     4.2.0            py39haa95532_0
appdirs                   1.4.4              pyhd3eb1b0_0
argon2-cffi               21.3.0             pyhd3eb1b0_0
argon2-cffi-bindings      21.2.0           py39h2bbff1b_0
arrow                     1.2.3                    pypi_0    pypi
asttokens                 2.0.5              pyhd3eb1b0_0
astunparse                1.6.3                    pypi_0    pypi
async-lru                 2.0.4            py39haa95532_0
attrs                     23.1.0           py39haa95532_0
babel                     2.11.0           py39haa95532_0
backcall                  0.2.0              pyhd3eb1b0_0
beautifulsoup4            4.12.3           py39haa95532_0
black                     23.7.0                   pypi_0    pypi
blas                      1.0                         mkl
bleach                    4.1.0              pyhd3eb1b0_0
bottleneck                1.3.7            py39h9128911_0
brotli                    1.0.9                h2bbff1b_8
brotli-bin                1.0.9                h2bbff1b_8
brotli-python             1.0.9            py39hd77b12b_8
brotlipy                  0.7.0           py39h2bbff1b_1003
bzip2                     1.0.8                h2bbff1b_6
ca-certificates           2025.11.12           h4c7d964_0    conda-forge
certifi                   2025.8.3           pyhd8ed1ab_0    conda-forge
cffi                      1.16.0           py39h2bbff1b_1
charset-normalizer        2.0.4              pyhd3eb1b0_0
click                     8.1.6                    pypi_0    pypi
colorama                  0.4.6            py39haa95532_0
comm                      0.2.1            py39haa95532_0
contourpy                 1.2.0            py39h59b6b97_0
cryptography              42.0.5           py39h89fc84f_1
cycler                    0.11.0             pyhd3eb1b0_0
debugpy                   1.6.7            py39hd77b12b_0
decorator                 5.1.1              pyhd3eb1b0_0
defusedxml                0.7.1              pyhd3eb1b0_0
entrypoints               0.4              py39haa95532_0
et_xmlfile                1.1.0            py39haa95532_0
exceptiongroup            1.2.0            py39haa95532_0
executing                 0.8.3              pyhd3eb1b0_0
fonttools                 4.51.0           py39h2bbff1b_0
fqdn                      1.5.1                    pypi_0    pypi
freetype                  2.12.1               ha860e81_0
giflib                    5.2.1                h8cc25b3_3
glib                      2.78.4               hd77b12b_0
glib-tools                2.78.4               hd77b12b_0
gst-plugins-base          1.22.3               h23ce68f_0
gstreamer                 1.22.3               h2bbff1b_0
icc_rt                    2022.1.0             h6049295_2
icu                       58.2                 ha925a31_3
idna                      3.7              py39haa95532_0
imageio                   2.37.0             pyhfb79c49_0    conda-forge
importlib-metadata        7.0.1            py39haa95532_0
importlib_metadata        7.0.1                hd3eb1b0_0
importlib_resources       6.1.1            py39haa95532_1
intel-openmp              2023.1.0         h59b6b97_46320
ipyflow                   0.0.178                  pypi_0    pypi
ipyflow-core              0.0.178                  pypi_0    pypi
ipykernel                 6.28.0           py39haa95532_0
ipympl                    0.9.3            py39haa95532_0
ipython                   8.15.0           py39haa95532_0
ipython_genutils          0.2.0              pyhd3eb1b0_1
ipywidgets                8.1.2            py39haa95532_0
isoduration               20.11.0                  pypi_0    pypi
jedi                      0.18.1           py39haa95532_1
jinja2                    3.1.4            py39haa95532_0
joblib                    1.4.2            py39haa95532_0
jpeg                      9e                   h2bbff1b_1
json5                     0.9.6              pyhd3eb1b0_0
jsonpointer               2.4                      pypi_0    pypi
jsonschema                4.19.2           py39haa95532_0
jsonschema-specifications 2023.7.1         py39haa95532_0
jupyter                   1.0.0            py39haa95532_9
jupyter-lsp               2.2.0            py39haa95532_0
jupyter_client            8.6.0            py39haa95532_0
jupyter_console           6.6.3            py39haa95532_0
jupyter_core              5.5.0            py39haa95532_0
jupyter_events            0.10.0           py39haa95532_0
jupyter_server            2.10.0           py39haa95532_0
jupyter_server_fileid     0.9.0            py39haa95532_0
jupyter_server_terminals  0.4.4            py39haa95532_1
jupyter_server_ydoc       0.8.0            py39haa95532_1
jupyter_ydoc              0.2.4            py39haa95532_0
jupyterlab                4.0.11           py39haa95532_0
```

jupyterlab_pygments	0.1.2	py_0	
jupyterlab_server	2.25.1	py39haa95532_0	
jupyterlab_widgets	3.0.10	py39haa95532_0	
kiwisolver	1.4.4	py39hd77b12b_0	
krb5	1.20.1	h5b6d351_0	
lcms2	2.12	h83e58a3_0	
lerc	3.0	hd77b12b_0	
libbrotlicommon	1.0.9	h2bbff1b_8	
libbrotlidec	1.0.9	h2bbff1b_8	
libbrotlienc	1.0.9	h2bbff1b_8	
libclang	14.0.6	default_hb5a9fac_1	
libclang13	14.0.6	default_h8e68704_1	
libdeflate	1.17	h2bbff1b_1	
libffi	3.4.4	hd77b12b_1	
libglib	2.78.4	ha17d25a_0	
libiconv	1.16	h2bbff1b_3	
libogg	1.3.5	h2bbff1b_1	
libpng	1.6.39	h8cc25b3_0	
libpq	12.17	h906ac69_0	
libsodium	1.0.18	h62dcd97_0	
libtiff	4.5.1	hd77b12b_0	
libvorbis	1.3.7	he774522_0	
libwebp	1.3.2	hbc33d0d_0	
libwebp-base	1.3.2	h2bbff1b_0	
libxml2	2.10.4	h0ad7f3c_2	
libxslt	1.1.37	h2bbff1b_1	
littleutils	0.2.4	pyhd8ed1ab_1	conda-forge
lxml	5.2.1	py39h09808a7_0	
lz4-c	1.9.4	h2bbff1b_1	
magpylib	5.1.1	pyhd8ed1ab_0	conda-forge
markupsafe	2.1.3	py39h2bbff1b_0	
matplotlib	3.8.4	py39haa95532_0	
matplotlib-base	3.8.4	py39h4ed8f06_0	
matplotlib-inline	0.1.6	py39haa95532_0	
mistune	2.0.4	py39haa95532_0	
mkl	2023.1.0	h6b88ed4_46358	
mkl-service	2.4.0	py39h2bbff1b_1	
mkl_fft	1.3.8	py39h2bbff1b_0	
mkl_random	1.2.4	py39h59b6b97_0	
mpmath	1.3.0	py39haa95532_0	
munkres	1.1.4	py_0	
mypy-extensions	1.0.0	pypi_0	pypi
narwhals	2.1.2	pyhe01879c_0	conda-forge
nbclassic	1.1.0	py39haa95532_0	
nbclient	0.8.0	py39haa95532_0	
nbconvert	7.10.0	py39haa95532_0	
nbformat	5.9.2	py39haa95532_0	
nest-asyncio	1.6.0	py39haa95532_0	
notebook	7.0.8	py39haa95532_0	
notebook-shim	0.2.3	py39haa95532_0	
numexpr	2.8.7	py39h2cd9be0_0	
numpy	1.26.4	py39h055cbcc_0	
numpy-base	1.26.4	py39h65a83cf_0	
openjpeg	2.4.0	h4fc8c34_0	
openpyxl	3.1.2	py39h2bbff1b_0	
openssl	3.6.0	h725018a_0	conda-forge
outdated	0.2.2	pyhd8ed1ab_2	conda-forge
overrides	7.4.0	py39haa95532_0	
packaging	23.2	py39haa95532_0	
pandas	2.2.2	py39h5da7b33_0	
pandas-flavor	0.6.0	pyhd8ed1ab_3	conda-forge
pandocfilters	1.5.0	pyhd3eb1b0_0	
parso	0.8.3	pyhd3eb1b0_0	
pathspec	0.11.2	pypi_0	pypi
patsy	0.5.6	py39haa95532_0	
pcre	8.45	hd77b12b_0	
pcre2	10.42	h0ff8eda_1	
pickleshare	0.7.5	pyhd3eb1b0_1003	
pillow	10.3.0	py39h2bbff1b_0	
pingouin	0.5.5	pyhd8ed1ab_1	conda-forge
pip	24.0	py39haa95532_0	
platformdirs	3.10.0	py39haa95532_0	
plotly	6.3.0	pyhd8ed1ab_0	conda-forge
ply	3.11	py39haa95532_0	
pmagpy	4.2.124	pypi_0	pypi
pooch	1.7.0	py39haa95532_0	
prometheus_client	0.14.1	py39haa95532_0	
prompt-toolkit	3.0.43	py39haa95532_0	
prompt_toolkit	3.0.43	hd3eb1b0_0	
psutil	5.9.0	py39h2bbff1b_0	
pure_eval	0.2.2	pyhd3eb1b0_0	
pybind11-abi	5	hd3eb1b0_0	
pyccolo	0.0.48	pypi_0	pypi
pycparser	2.21	pyhd3eb1b0_0	
pygments	2.15.1	py39haa95532_1	
pyopenssl	24.0.0	py39haa95532_0	
pyparsing	3.0.9	py39haa95532_0	

```
pyqt                      5.15.10          py39hd77b12b_0
pyqt5-sip                 12.13.0          py39h2bbff1b_0
pyrsistent                0.20.0           py39h2bbff1b_0
pysocks                   1.7.1            py39haa95532_0
python                    3.9.18               h1aa4202_0
python-dateutil           2.9.0post0       py39haa95532_2
python-fastjsonschema     2.16.2           py39haa95532_0
python-json-logger        2.0.7            py39haa95532_0
python-tzdata             2023.3             pyhd3eb1b0_0
pytz                      2024.1           py39haa95532_0
pywin32                   305              py39h2bbff1b_0
pywinpty                  2.0.10           py39h5da7b33_0
pyyaml                    6.0.1            py39h2bbff1b_0
pyzmq                     25.1.2           py39hd77b12b_0
qt-main                   5.15.2               h879a1e9_9
qt-webengine              5.15.9               h5bd16bc_7
qtconsole                 5.5.1            py39haa95532_0
qtpy                      2.4.1            py39haa95532_0
qtwebkit                  5.212                h2bbfb41_5
referencing               0.30.2           py39haa95532_0
requests                  2.32.2           py39haa95532_0
rfc3339-validator         0.1.4            py39haa95532_0
rfc3986-validator         0.1.1            py39haa95532_0
rpds-py                   0.10.6           py39h062c2fa_0
scikit-learn              1.5.1            py39hc64d2fc_0
scikit-posthocs           0.11.2             pyhd8ed1ab_1    conda-forge
scipy                     1.13.1           py39h8640f81_0
seaborn                   0.13.2           py39haa95532_0
seaborn-base              0.13.2             pyhd8ed1ab_0    conda-forge
send2trash                1.8.2            py39haa95532_0
setuptools                69.5.1           py39haa95532_0
sip                       6.7.12           py39hd77b12b_0
six                       1.16.0             pyhd3eb1b0_1
sniffio                   1.3.0            py39haa95532_0
soupsieve                 2.5              py39haa95532_0
sqlite                    3.45.3               h2bbff1b_0
stack_data                0.2.0              pyhd3eb1b0_0
statsmodels               0.14.2           py39h827c3e9_0
tabulate                  0.9.0            py39haa95532_0
tbb                       2021.8.0             h59b6b97_0
terminado                 0.17.1           py39haa95532_0
threadpoolctl             3.5.0            py39h9909e9c_0
tinycss2                  1.2.1            py39haa95532_0
tk                        8.6.14               h0416ee5_0
toml                      0.10.2             pyhd3eb1b0_0
tomli                     2.0.1            py39haa95532_0
tornado                   6.4.1            py39h827c3e9_0
traitlets                 5.14.3           py39haa95532_0
typing-extensions         4.11.0           py39haa95532_0
typing_extensions         4.11.0           py39haa95532_0
tzdata                    2024a                h04d1e81_0
ucrt                      10.0.22621.0         h57928b3_0    conda-forge
unicodedata2              15.1.0           py39h2bbff1b_0
uri-template              1.3.0                    pypi_0    pypi
urllib3                   2.2.1            py39haa95532_0
vc                        14.3                h2b53caa_32    conda-forge
vc14_runtime              14.44.35208        h818238b_32    conda-forge
vcomp14                   14.44.35208        h818238b_32    conda-forge
vs2015_runtime            14.44.35208        h38c0c73_32    conda-forge
wcwidth                   0.2.5              pyhd3eb1b0_0
webcolors                 1.13                     pypi_0    pypi
webencodings              0.5.1            py39haa95532_1
websocket-client          1.8.0            py39haa95532_0
wheel                     0.43.0           py39haa95532_0
widgetsnbextension        4.0.10           py39haa95532_0
win_inet_pton             1.1.0            py39haa95532_0
winpty                    0.4.3                         4
xarray                    2023.6.0         py39haa95532_0
xlrd                      2.0.1              pyhd3eb1b0_1
xz                        5.4.6                h8cc25b3_1
y-py                      0.5.9            py39hb6bf4ef_0
yaml                      0.2.5                he774522_0
ypy-websocket             0.8.2            py39haa95532_0
zeromq                    4.3.5                hd77b12b_0
zipp                      3.17.0           py39haa95532_0
zlib                      1.2.13               h8cc25b3_1
zstd                      1.5.5                hd43e919_2

Note: you may need to restart the kernel to use updated packages.
```

In [ ]:
